# Identification of Unstable Network Modules Reveals Disease Modules Associated with the Progression of Alzheimer’s Disease

**DOI:** 10.1371/journal.pone.0076162

**Published:** 2013-11-15

**Authors:** Masataka Kikuchi, Soichi Ogishima, Tadashi Miyamoto, Akinori Miyashita, Ryozo Kuwano, Jun Nakaya, Hiroshi Tanaka

**Affiliations:** 1 Department of Bioinformatics, Tokyo Medical and Dental University, Bunkyo-ku, Tokyo, Japan; 2 Department of Bioclinical Informatics, Tohoku Medical Megabank Organization, Tohoku University, Sendai-shi, Miyagi, Japan; 3 Bioresource Science Branch, Center for Bioresources, Brain Research Institute, Niigata University, Niigata-shi, Niigata, Japan; Semmelweis University, Hungary

## Abstract

Alzheimer’s disease (AD), the most common cause of dementia, is associated with aging, and it leads to neuron death. Deposits of amyloid β and aberrantly phosphorylated tau protein are known as pathological hallmarks of AD, but the underlying mechanisms have not yet been revealed. A high-throughput gene expression analysis previously showed that differentially expressed genes accompanying the progression of AD were more down-regulated than up-regulated in the later stages of AD. This suggested that the molecular networks and their constituent modules collapsed along with AD progression. In this study, by using gene expression profiles and protein interaction networks (PINs), we identified the PINs expressed in three brain regions: the entorhinal cortex (EC), hippocampus (HIP) and superior frontal gyrus (SFG). Dividing the expressed PINs into modules, we examined the stability of the modules with AD progression and with normal aging. We found that in the AD modules, the constituent proteins, interactions and cellular functions were not maintained between consecutive stages through all brain regions. Interestingly, the modules were collapsed with AD progression, specifically in the EC region. By identifying the modules that were affected by AD pathology, we found the transcriptional regulation-associated modules that interact with the proteasome-associated module via UCHL5 hub protein, which is a deubiquitinating enzyme. Considering PINs as a system made of network modules, we found that the modules relevant to the transcriptional regulation are disrupted in the EC region, which affects the ubiquitin-proteasome system.

## Introduction

The most common cause of dementia is late-onset Alzheimer’s disease (AD), which is associated with age > 65 years and leads to neuron death. The AD brain is characterized by atrophy, which is measured using volumetric magnetic resonance imaging (MRI). Postmortem, the AD brain shows senile plaques on the surface of the cerebral neocortex and neurofibrillary tangle (NFT) staining. Senile plaques are deposits of amyloid beta protein (Aβ) spliced out by cleavage of the amyloid precursor protein (APP). NFTs are aggregations of aberrantly phosphorylated microtubule-associated protein tau (MAPT), a protein that lets microtubules stabilize in general. The deposit of NFTs expands from the central regions of the brain (e.g., entorhinal cortex, hippocampus) to the neocortex. This pathological stage of AD is defined by Braak stages. Braak stages are described as transentorhinal stages (Braak stages I‒II), limbic stages (Braak stages III‒IV) and isocortical stages (Braak stages V‒VI) [1].

 To elucidate the mechanisms of the pathogenesis and progression of AD, high-throughput gene expression analyses using DNA microarrays have been conducted; in postmortem AD brains, differentially expressed genes associated with AD progression have been found to be more down-regulated than up-regulated in the later stages of AD (Braak stage, density of cerebrocortical neuritic plaque and clinical dementia rating scale [CDR] that is a scale to measure the severity of dementia) [2]. Even in normal postmortem brains, gene expression profiles change with age and differ between males and females [3,4]. However, it has not yet been determined whether the molecular networks in the various brain regions can be affected by these changes of gene expression profiles during the progression of AD and normal aging.

 Understanding the dynamics of the molecular networks that accompany the progression of AD can lead to the development of biomarkers for this disease [5] and help to elucidate the mechanisms of the pathogenesis and progression of AD. Barabasi et al. hypothesized that in disease, modules are disrupted into “disease modules” due to mutations, deletions, copy number aberrations (CNAs), and expression aberrations [6]. Disease modules are considered to lose their original network structures and their original functions during disease progression. In AD, analyses of co-expression networks and the crosstalk of pathways have offered some insights into the mechanisms of the pathogenesis and progression of AD [7–9]. However, the following questions have yet to be answered. How are the molecular networks disrupted with AD progression in brain regions? What are the disease modules in AD?

 To uncover how the molecular networks and their constituent modules collapse into dysfunction during AD progression, we here show in detail (1) the disruption of protein expressions, interactions, and protein interaction networks (PINs) (2), the instability of modules and increasing dysfunction with AD progression, and (3) AD-disrupted modules—i.e., disease modules— that can help elucidate the mechanisms underlying the pathogenesis and progression of AD.

## Results and Discussion

### Overview of this study

To uncover how the molecular networks and their constituent modules are disrupted with the progression of AD, we used gene expression profiles of AD brains and healthy brains from a public gene expression database and a human protein-protein interaction database (see Materials and Methods).

 Gene expression profiles were obtained from healthy-brain subjects (accession number: GSE11882) and from AD-brain subjects (GSE5281) in three brain regions: the entorhinal cortex (EC), hippocampus (HIP) and superior frontal gyrus (SFG). The EC and the HIP belong to the limbic system, and connect with each other through the perforant pathway. These two regions are associated with short-term and long-term memory as well as spatial memory [10,11]. The SFG is part of the frontal lobe, and it contributes to working memory [12]. In AD, the EC and the HIP are affected in the early stage, and the SFG is affected in the later stages.

 The gene expression profiles of the healthy brains were from subjects who were 20 to 99 years old. Among them, we considered the healthy brains of subjects over 60 years old as normal-aging brains, because late-onset Alzheimer’s disease (i.e., sporadic AD without genetic causes) is known to affect individuals over 65 years old [13–15]. Normal aging subjects were classified into the following four age groups: 60–69, 70–79, 80–89, and 90–99 y/o. Similarly, AD datasets were also grouped into four Braak stages. The EC datasets were classified into the Braak stage I, II, III and IV because Braak stages V and VI are not applicable to the EC. In contrast, the HIP and the SFG datasets were classified into Braak stage I, II, V and VI since Braak stages III and IV are inapplicable to the HIP and the SFG. Note that AD and normal aging in each brain region were classified into the four stages or groups.

**Figure 1:**
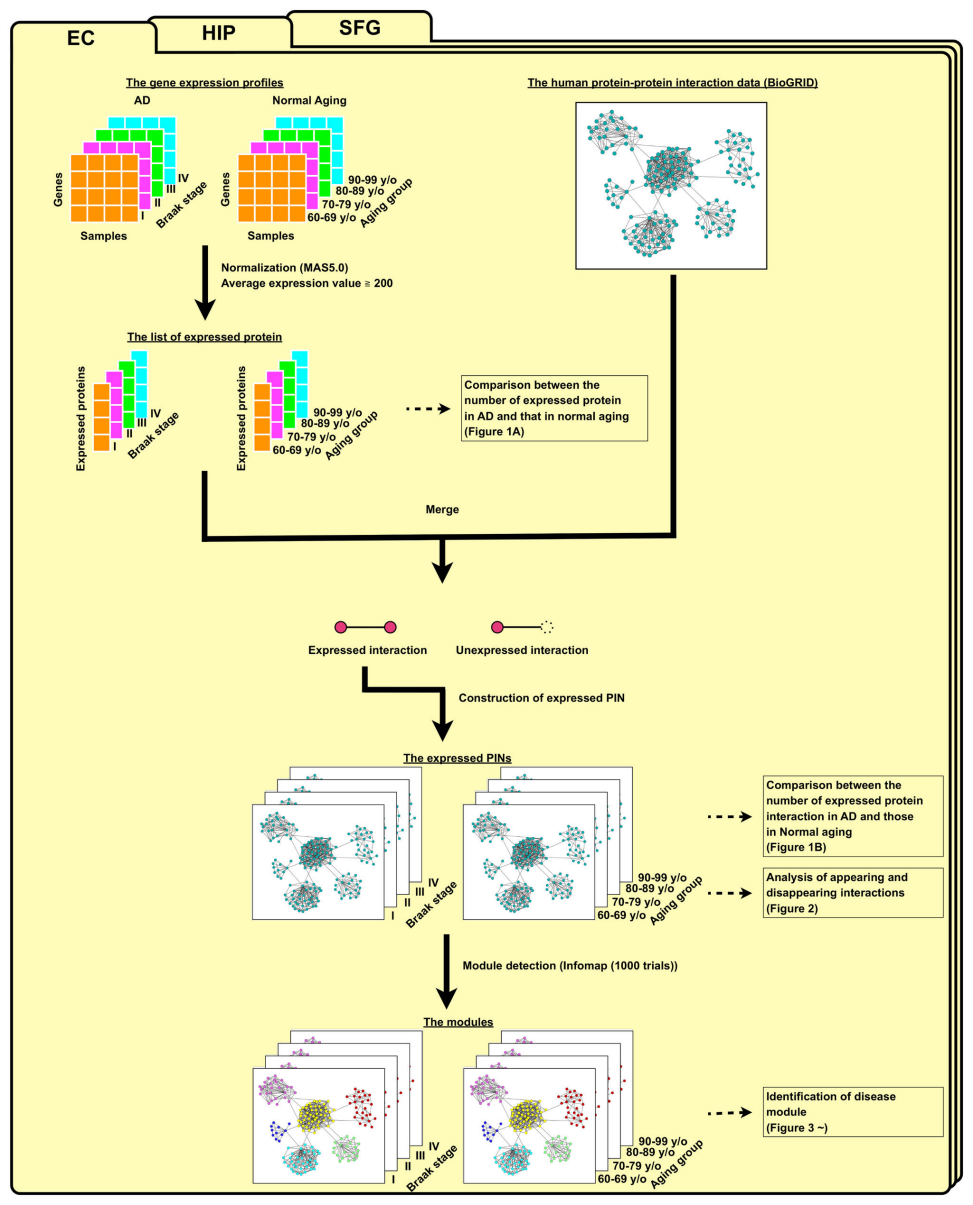
Flowchart for the identification of expressed protein interaction networks (PINs) and the detection of module. Expressed proteins were extracted from gene expression profiles based on our criteria: detection call is “present” and the average expression value is more than 200. Merging the list of expressed proteins and protein-protein interaction data, we obtained interactions whose constituent proteins were expressed at the same time as expressed protein interactions, and we constructed expressed PINs. We then detected modules from expressed PINs by using the Infomap algorithm. These processes were also performed in the other brain regions.

 We analyzed gene expression profiles of the AD and normal aging brains according to our workflow, shown in Figure 1. First, we normalized gene expression datasets using the MAS 5.0 algorithm (Affymetrix, Santa Clara, CA). For each probe set, the average expression values were calculated using the samples marked as “present” by the detection call algorithm (Affymetrix). We considered that a gene was expressed if the average expression values exceeded 200 [10,11].

 To characterize the disruption of PINs in AD, we then studied genome-wide changes of PINs in AD from the following three levels: (1) individual proteins, (2) pairs of known interacting proteins, and (3) sets of proteins we called “modules.” Among the protein-protein interactions from the BioGRID (Release 3.1.84) [16,17], we identified expressed protein interactions whose constituent proteins were expressed at the same time [18,19]. Expressed protein interactions were assembled into an “expressed PIN” in each Braak stage and each age group. To divide the expressed PINs into modules, we used the Infomap algorithm [20,21].

### Disruption of expressions of protein interactions in AD

To examine the disruption of protein interactions in AD, we identified the expressed proteins and their interactions, and then examined their numbers in the normal aging and AD groups. A protein was hypothesized to be expressed if the corresponding gene was expressed. An expressed protein was thus defined as a protein if the corresponding gene was expressed, and an expressed protein interaction was thus defined as a protein interaction whose constituent proteins were expressed at the same time. We identified expressed protein interactions in each brain region (EC, HIP and SFG) in each age group and AD progression stage, and then collected the expressed protein interactions as an expressed protein interaction network (PIN) in each brain region for each age group and AD progression stage.

**Figure 2:**
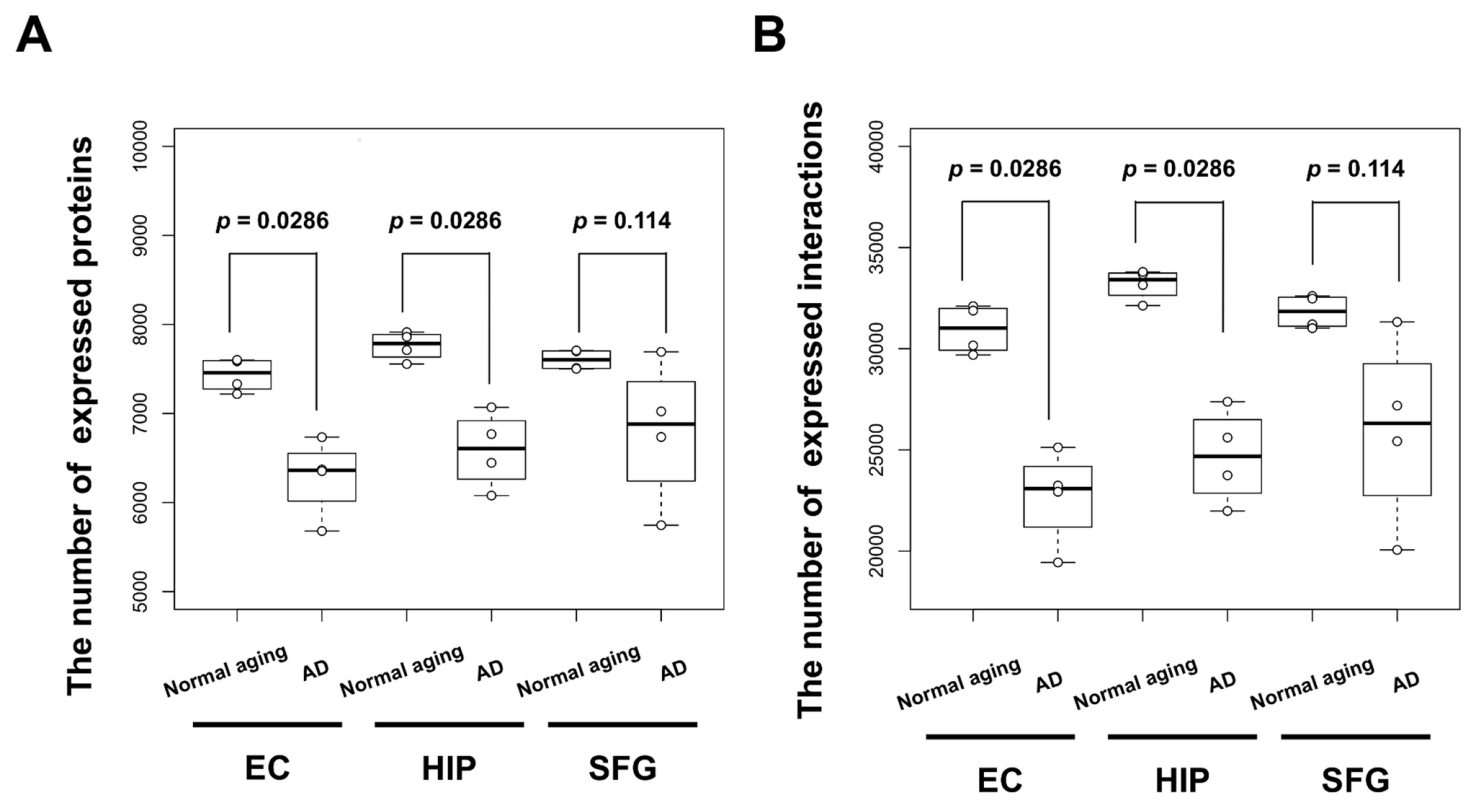
The number of expressed proteins and interactions in expressed PINs in normal aging and AD. A boxplot represents the numbers of (A) expressed proteins or (B) expressed interactions for normal age groups (60‒69, 70‒79, 80‒89, 90‒99 years old) and AD progression stages (Braak stage I, II, III/IV, V/VI). The numbers of expressed proteins and interactions in the AD EC and HIP were significantly lower than those in the normal-aging groups (Wilcoxon test; P-value = 0.0286, respectively). Four samples in AD and four samples in normal aging were compared. In the SFG, the number of expressed interactions in AD was not significantly lower than that in normal aging (Wilcoxon test; P-value = 0.114).

 We compared the numbers of expressed proteins and interactions in AD with those in normal aging. We found that these numbers in AD were significantly lower than those in normal aging across the EC and HIP regions (Wilcoxon test; *P*-value = 0.0286; Figure 2A,B). The EC and HIP regions were affected by AD from the incipient stages of AD pathogenesis; protein expressions and interactions in the AD EC and AD HIP regions were also thought to be disrupted from the beginning of AD pathogenesis.

### Disruption of PINs along with AD progression

As described above, the AD PINs were smaller than the normal-aging PINs. To what extent were the AD cellular networks disrupted with the progression of AD?

We examined correlations of the gene expression levels of proteins that appear and disappear with aging and AD progression (Figures S1 and S2). The all expression levels showed significantly correlated with both aging and AD progression in each brain region. 

**Figure 3:**
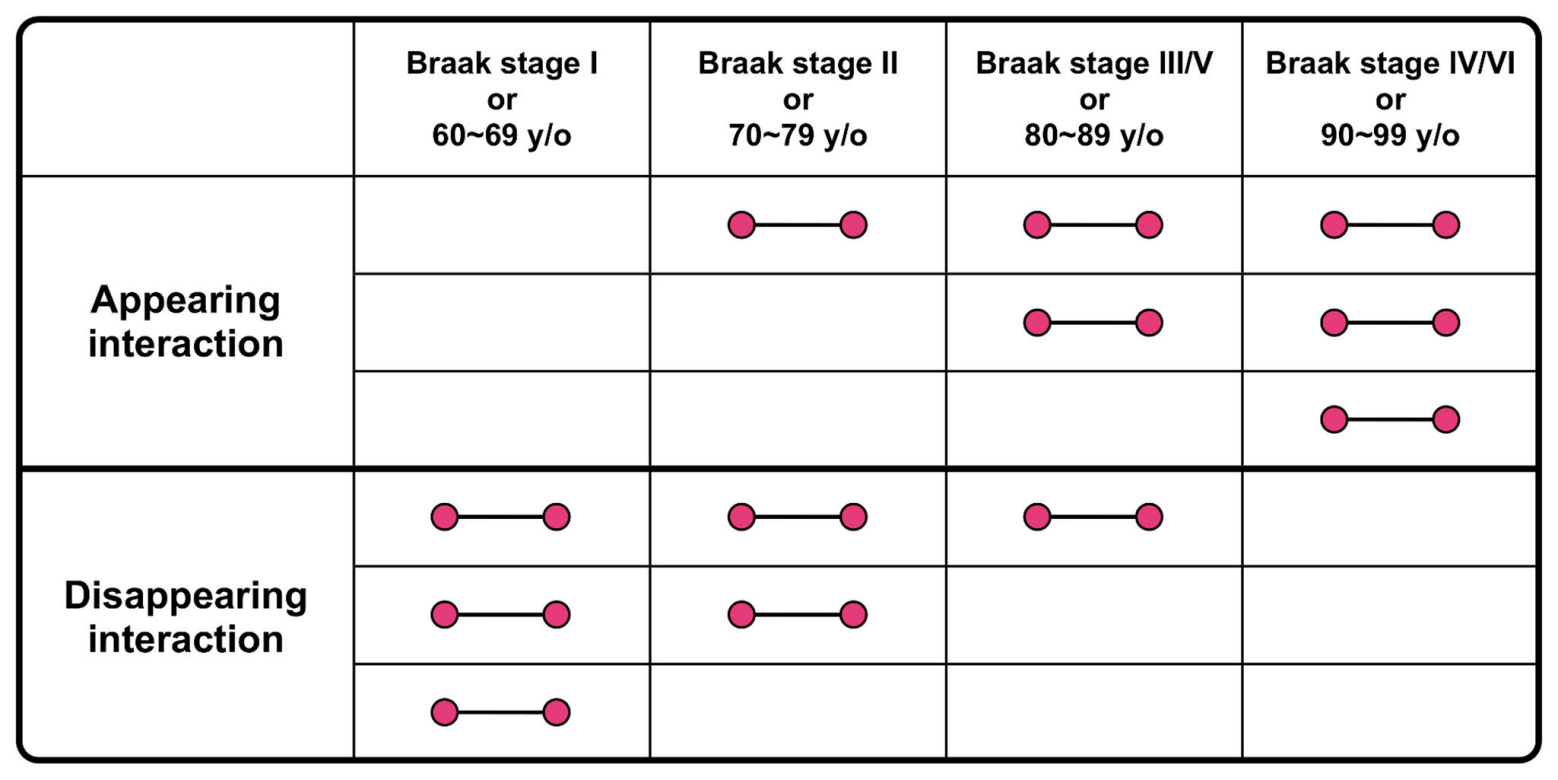
A scheme of appearing and disappearing interactions. An appearing interaction was defined as an expressed protein interaction that was not expressed at an early age or stage of AD progression but was expressed in later stages and age groups. A disappearing interaction was defined as an expressed protein interaction that was expressed at an early age or AD progression stage but was not expressed in later stages or age groups. Each interaction has three patterns indicated in a scheme.

Furthermore, we identified the appearing and disappearing interactions with aging and AD progression in each brain region (Figure 3). An appearing interaction was defined as an expressed protein interaction that was not expressed at an early age or stage of AD progression but was expressed in later stages and age groups. A disappearing interaction was defined as an expressed protein interaction that was expressed at an early age or AD stage but was not expressed in later stages or age groups.

**Figure 4:**
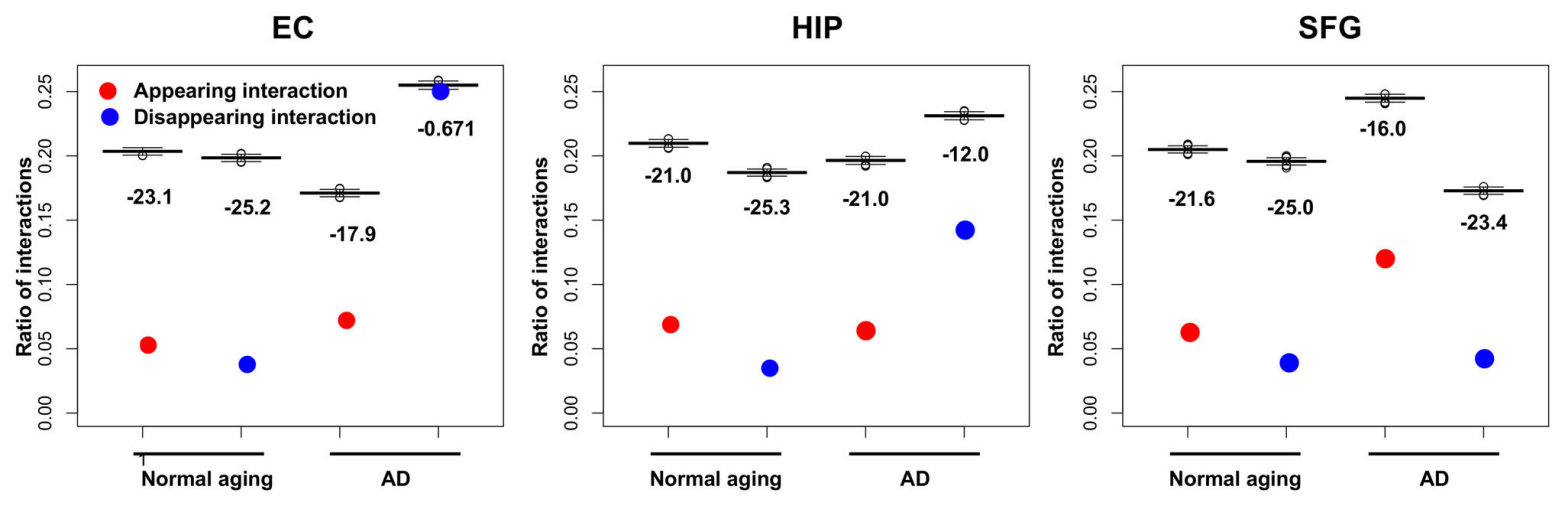
Ratios of appearing and disappearing interactions. Red plots indicate the ratios of appearing protein interactions, and blue plots indicate disappearing protein interactions. The boxplots indicate the ratios of appearing and disappearing protein interactions from 1,000 corresponding randomized networks in each brain region in normal aging and AD. Values below the boxplots show the Z-scores between the ratio and the ratios of 1,000 randomized networks. The ratio of the number of disappearing interactions in the AD entorhinal cortex (EC) region showed no significant difference from those of the 1,000 randomized networks (Z-score = −0.671).

 We compared the ratios of appearing and disappearing interactions to all expressed protein interactions in AD and normal aging with those in randomized networks composed of interactions whose number was equal to the number of expressed interactions from all protein interactions without self-interactions retrieved from the BioGRID (see details for Material and Methods)(Figure 4). Except for the EC region in the AD brains, the ratios of appearing and disappearing interactions to all expressed protein interactions in the three regions of both the AD and normal-aging brains were significantly and remarkably lower than those in the corresponding randomized networks, resulting in Z-scores between −26.5 and −12.0, which suggested that the appearance and disappearance of interactions were significantly suppressed in age groups and AD progression stages (except for the AD EC region) compared to the corresponding randomized networks.

Interestingly, the ratio of disappearing interactions in the AD EC region was not significantly lower than those in its randomized networks (Z-score = −0.672), which suggested that disappearance of interactions was no longer suppressed in the AD EC region compared with its randomized networks. Therefore, the AD EC region lost the original functions of its PINs, and it lost protein interactions along with the progression of AD, which resulted in disruption and dysfunction of its PINs.

### Instability of consecutive modules in PINs during AD

**Figure 5:**
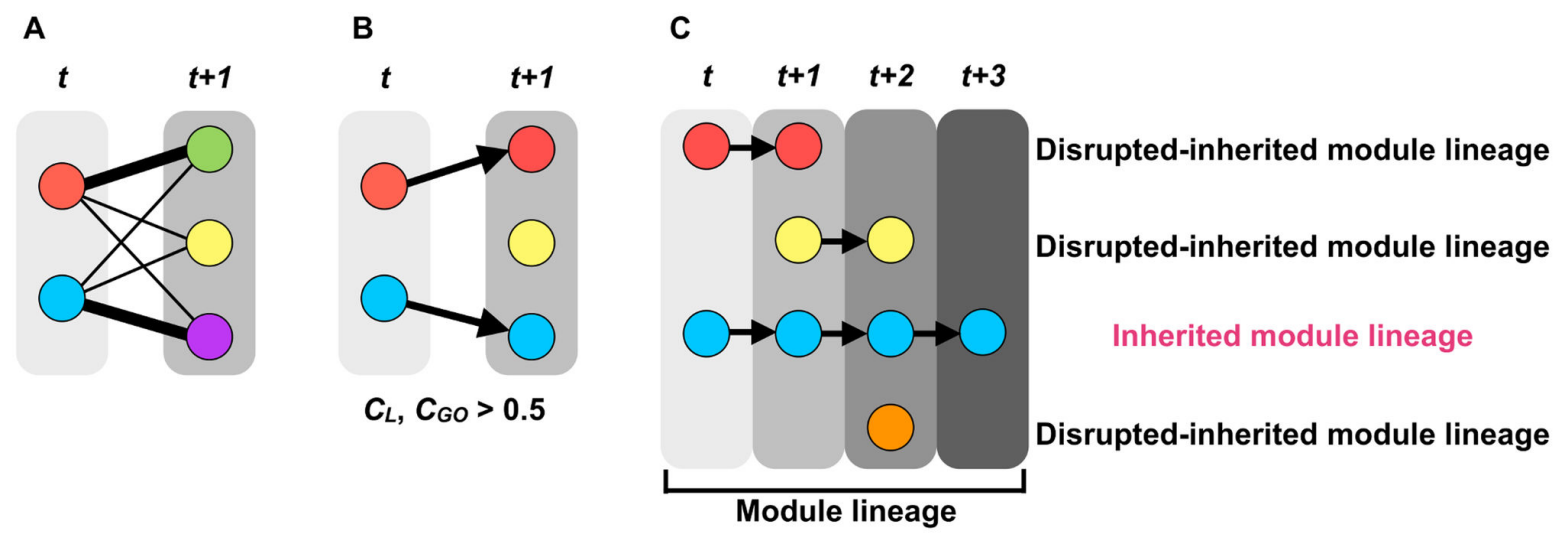
The process used to generate the module lineages. (A) Calculation of the interactions (C_L_) of all possible module pairs in two consecutive stages. (B) Of the module pairs exhibiting the highest *C*_*L*_, if *C*_*L*_ and *C*_*GO*_ exceeded 0.5, the modules were considered “inherited.” (C) If the modules were inherited from the earliest age group or AD progression stage (60–69 y/o or Braak stage I) to the latest age group or AD progression stage (90–99 y/o or Braak stage IV in the EC region or Braak stage VI in the HIP and the SFG regions), we called these modules “inherited-module lineages,” and called the other modules “disrupted inherited-module lineages.” Each node indicates distinct modules. Thick links are the module pairs exhibiting the highest CL. Arrows represent inherited relationships.

**Figure 6:**
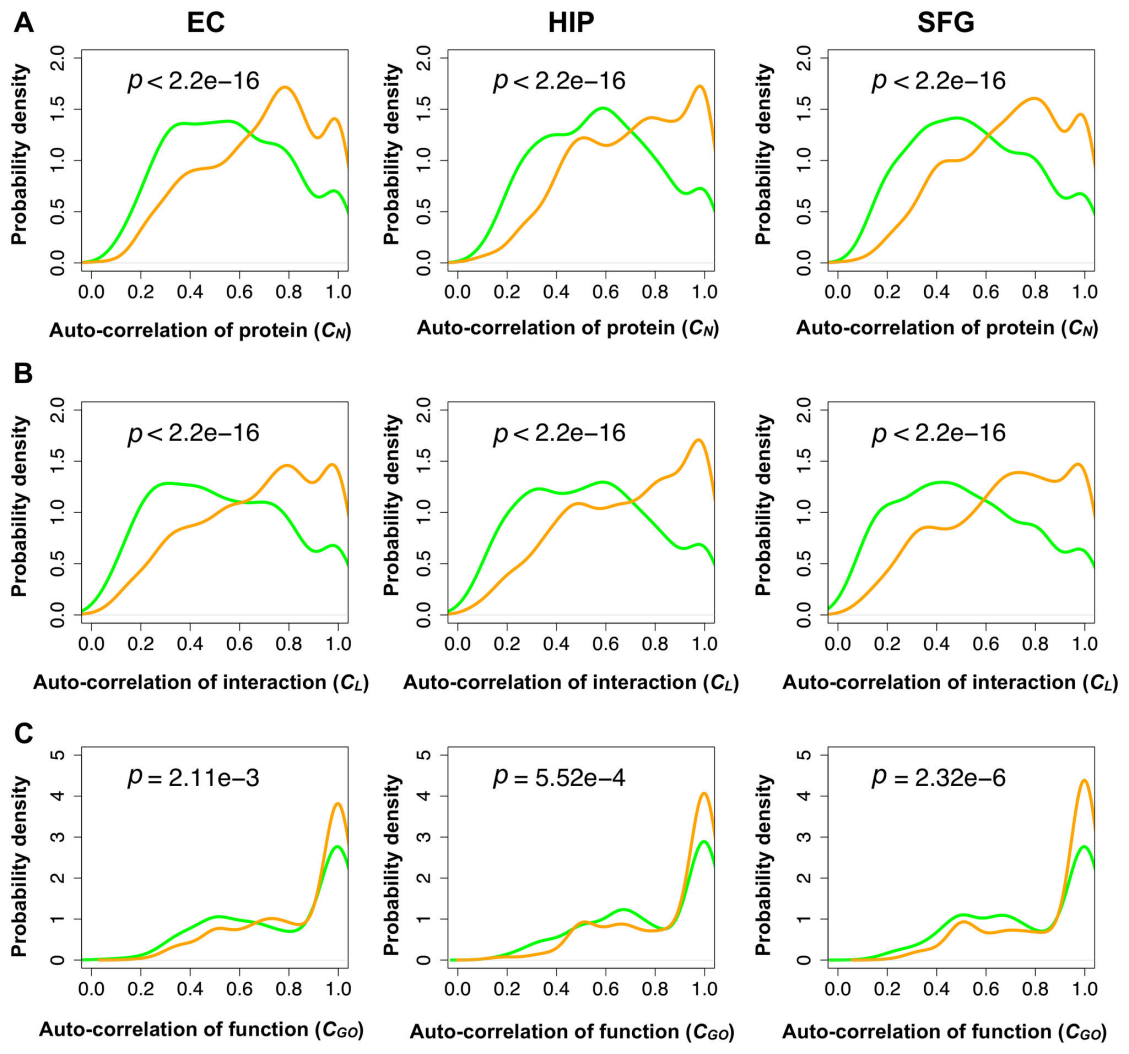
Auto-correlations of proteins, interactions, and functions for inherited modules. Probability density distributions of the (A) auto-correlation of proteins, (B) interactions, and (C) cellular functions of a consecutive module pair. Orange curves indicate normal aging and green curves indicate AD. P-values were calculated from the Wilcoxon test. Auto-correlations in AD were significantly lower than those in normal aging through all brain regions.

To clarify the disruption of PINs during AD, we traced “modules” composed of their expressed PINs along the progression of AD and aging. We divided the expressed PINs in aging and AD into modules based on the network structure, using the Infomap algorithm [20,21]. The Infomap algorithm is known for showing superior performance [22]. In our previous study, we showed that the Infomap algorithm has high Q-modularity, which is a quality index for divisions of a network [23]; the use of this algorithm finely divided the PINs into modules compared to the other methods (Table S1). As a result, 309–392 modules were detected in expressed PINs in each brain region of each age group and each AD progression stage. The detected modules were then tracked between consecutive age groups and AD progression stages though aging and AD progression. We assessed the stability of modules by the auto-correlation of proteins (*C*_*N*_), interactions (*C*_*L*_), and cellular functions (*C*_*GO*_). Regarding each auto-correlation, among all the possible pairs of tracked modules between consecutive age groups or AD progression stages, the module pairs exhibiting the highest auto-correlation were identified (Figure 5A). We also obtained the probability density distributions of *C*_*N*_, *C*_*L*_, and *C*_*GO*_ in each brain region for each age group and AD progression stage (Figure 6).

 As shown in Figure 6, across all the brain regions, the module pairs in the normal-aging brains showed significantly higher *C*_*N*_, *C*_*L*_, and *C*_*GO*_ values compared to their counterparts in the AD brains. Most of the modules in the consecutive age groups maintained their constituent proteins, interactions and functions, whereas most modules in the consecutive AD progression groups dynamically changed their constituent proteins, interactions and functions, suggesting a dysfunction of modules.

### Few inherited-module lineages along with AD progression

If a module pair exhibits the highest *C*_*L*_ in two consecutive stages and their *C*_*L*_ and *C*_*GO*_ exceeded 0.5 (i.e., over one-half), we assumed that the modules were “inherited” (Figure 5B). If the modules were inherited from the earliest age group or AD progression stage (60–69 y/o or Braak stage I) to the latest age group or AD progression stage (90–99 y/o or Braak stage IV in the EC region or Braak stage VI in the HIP and the SFG regions), we called these modules “inherited-module lineages” and called the other modules “disrupted inherited-module lineages” (Figure 5C).

**Figure 7:**
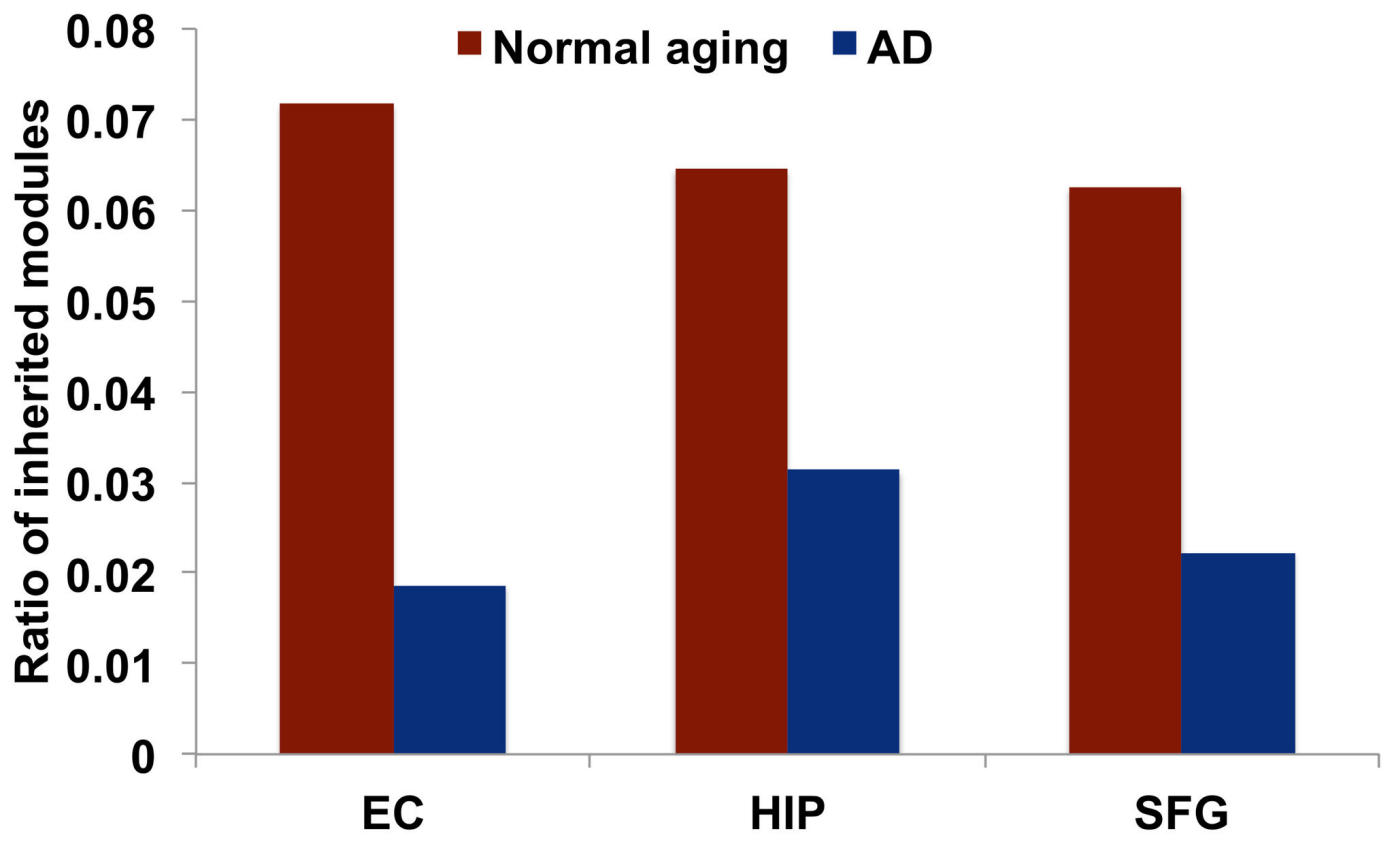
Ratio of inherited module lineages. The ratio of inherited module lineages to the total number of module lineages. The ratios of inherited module lineages in AD were lower than those in normal aging through all brain regions.

 Accordingly, we identified 1046–1212 module lineages (including inherited-module lineages and disrupted inherited-module lineages). Inherited-module lineages imply modules that have a stable network structure and maintain their cellular functions with aging and AD progression. As a result, in the normal-aging brains, 7.17% (75/1046), 6.46% (69/1069), and 6.25% (68/1088) module lineages were identified as inherited-module lineages in the EC, HIP and SFG regions, respectively (Figure 7). In AD, 1.87% (21/1123), 3.13% (35/1118), and 2.23% (27/1212) of the module lineages were identified as inherited-module lineages in the EC, HIP and SFG regions, respectively. The results held using different thresholds (that is, *C*_*L*_ and *C*_*GO*_ exceeded 0.3, 0.4, 0.6, 0.7) (Figure S3). As shown above, the ratio of inherited-module lineages to all module lineages in AD was less than that in normal aging. Thus, stable inherited-module lineages were fewer in AD; stated differently, disrupted inherited-module lineages were relatively abundant in AD.

 In addition, we compared among module sizes of inherited module lineages and appearing/disappearing module lineages (Figure S4 and Table S2). As a result, module sizes of inherited module lineages were significantly ~2.2-fold higher than them of appearing/disappearing module lineages. On the other hand, we did not find differences of module sizes between appearing and disappearing module lineages. These results suggest that aging and disease progression allow variances of small modules rather than them of large modules.

### Disease modules along with AD progression

In the previous section, we noted that the ratios of inherited module lineages in AD were less than those in normal aging, meaning that the module lineages in AD are unstable and dynamic. However, the module lineages in normal aging were not always inherited-module lineages. This finding suggests that the stability of a module is affected not only by AD progression but also by aging. To uncover disease modules from identified module lineages, we needed to compare module lineages between normal aging and AD. We then examined the correspondence between 60–69 y/o and Braak stage I, 70–79 y/o and Braak stage II, 80–89 y/o and Braak stage III (in the EC) or V (in the HIP and SFG), and 90–99 y/o and Braak stage IV (in the EC) or VI (in the HIP and SFG).

 To find the correspondence of module lineages between normal aging and AD, we aligned their constituent modules at each age group and each AD progression stage (e.g., a module in 60–69 y/o and a module in Braak stage I). We then evaluated the correspondence between aligned modules by calculating the auto-correlation of their interactions (*C*_*L*_) and cellular functions (*C*_*GO*_). If an aligned module pair exhibited the highest *C*_*L*_ and both *C*_*L*_ and *C*_*GO*_ were over 0.5, the aligned modules showed a correspondence of both constituent interactions and exhibited functions.

 If a module lineage in normal aging is an inherited module lineage and the corresponding module lineage in AD is disrupted, we can assume that the module lineage collapses with AD progression. We called such modules “AD-disrupted modules.”

**Figure 8:**
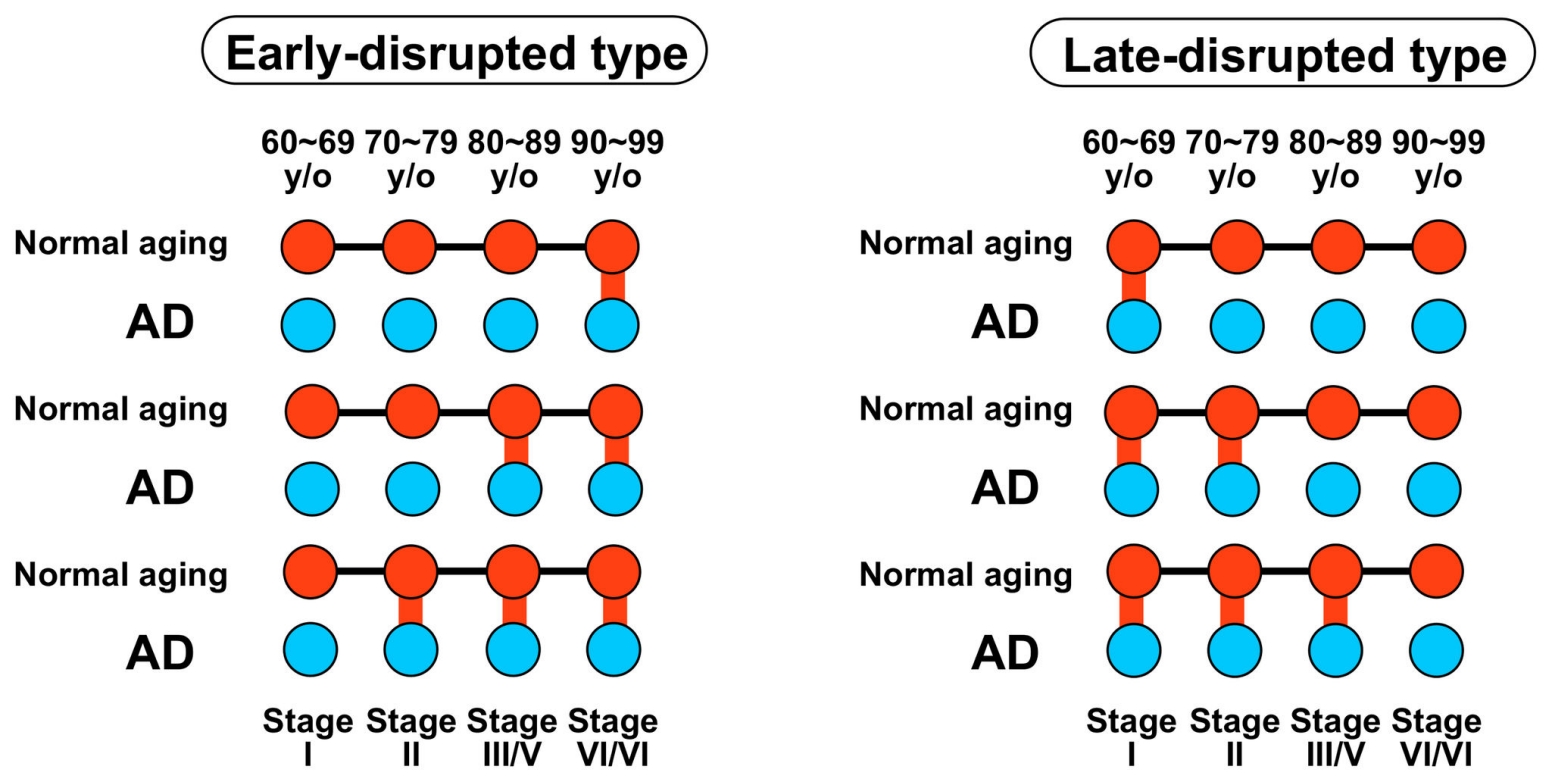
Schematic illustration of AD-disrupted modules. AD-disrupted modules were classified as the early-disrupted type or the late-disrupted type. Red nodes: modules in normal aging. Blue nodes: modules in AD. Black lines: inherited relationships in normal aging. Red lines: correspondences between the module in each age group in normal aging and the module in the corresponding Braak stage in AD.

 Each AD-disrupted module was classified as either the early-disrupted type or the late-disrupted type (Figure 8 and Table S3). An early-disrupted type was defined as a module in an AD brain that has no correspondence to an early age group in normal aging, but corresponds to a later age group. A late-disrupted type was defined as a module in AD that corresponds to an early age group in normal aging, but has no correspondence to a later age group.

 The ratio of the number of late-disrupted types to the number of inherited module lineages of normal aging in the EC region was 40.0%, and was higher than those in the other regions (3.45 times that in the HIP region, 3.88 times that in the SFG region) (Table 1). This finding is consistent with the result shown in Figure 4 that the ratio of disappearing interactions was equivalent to that in randomized networks in the AD EC region.

**Table 1:** The number of module lineages in early- and late-disrupted types.

	**Module type**			
**Brain region**	**Early-disrupted type**	**Late-disrupted type**	**Other**	**Total (All inherited module lineages)**
**EC**	**3 (4.0%)**	**30 (40.0%)**	**42 (56.0%)**	**75**
**HIP**	**3 (4.3%)**	**8 (11.6%)**	**58 (84.1%)**	**69**
**SFG**	**2 (2.9%)**	**7 (10.3%)**	**59 (86.8%)**	**68**

Inherited module lineages in normal aging were divided into two module types (early- and late-disrupted type). The numbers in parentheses represent the ratios of the number of module lineages to the number of all corresponding inherited module lineages.

**Figure 9:**
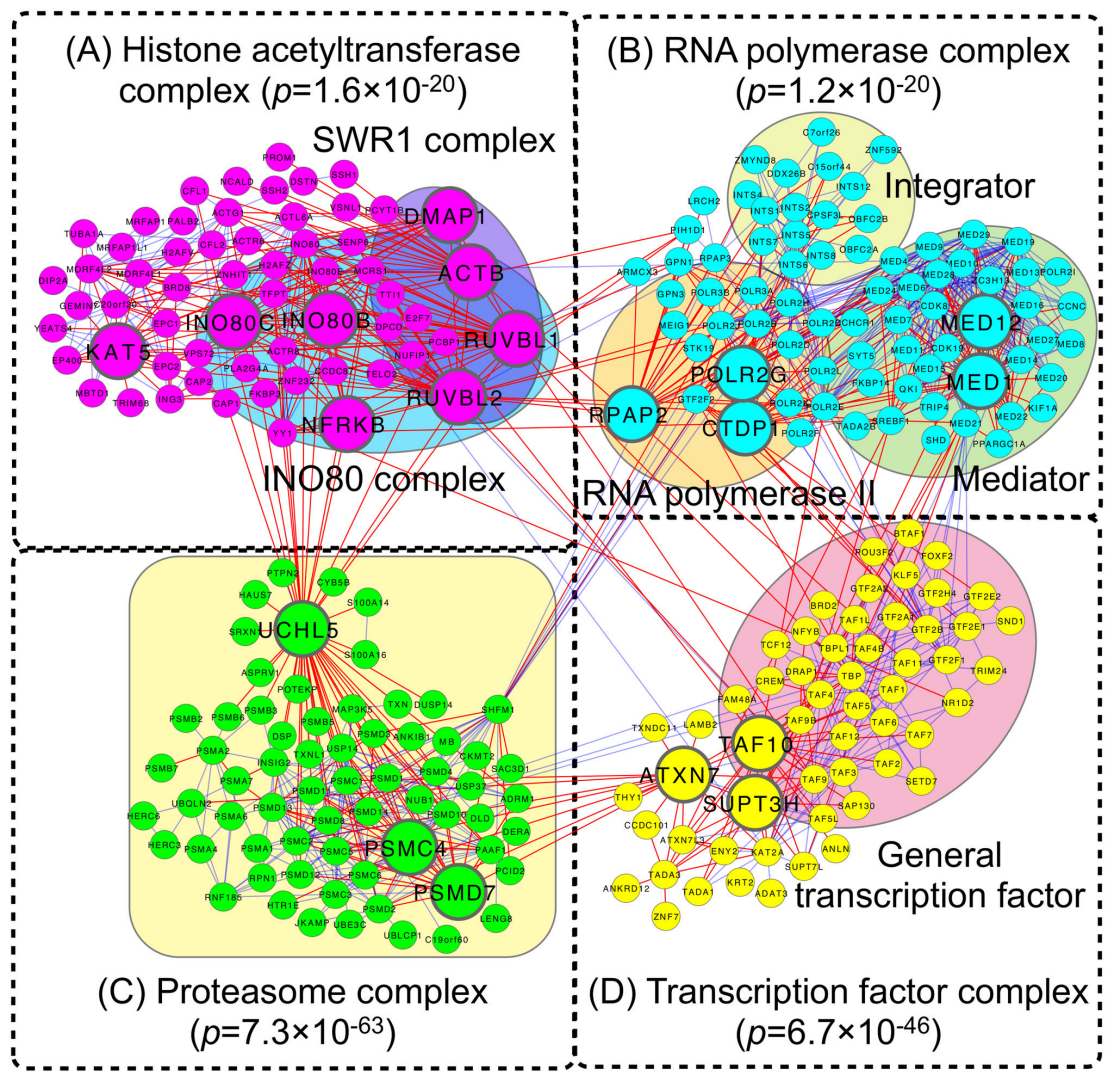
Late-disrupted modules in the EC region. (A) The histone acetyltransferase-associated module, which is the late-disrupted module with the largest number of disappearing interactions. (B) The RNA polymerase-associated module, which is the late-disrupted module with the second largest number of disappearing interactions. (C) The proteasome-associated module, which is the late-disrupted module sharing the largest number of disappearing interactions with the histone acetyltransferase-associated module. (D) The transcription factor-associated module is the late-disrupted module sharing the largest number of disappearing interactions with the RNA polymerase-associated module. The large nodes are hub proteins with more than 10 disappearing interactions in the module. Links in red and blue indicate disappearing interactions and the other, respectively. These modules are depicted by the superimposition of modules in AD that corresponded with normal aging.

 We then identified the two late-disrupted modules in the EC region with the largest and second-largest numbers of disappearing interactions (Figure 9A and B, respectively). A gene ontology (GO) analysis using the DAVID algorithm [24,25] revealed that the modules in Figure 9A and 9B were associated with histone acetyltransferase complex and RNA polymerase complex, respectively (modified Fisher’s exact test; *P*-value = 1.6×10^−20^ and 1.2×10^−20^, respectively).

 Regarding the histone acetyltransferase-associated module, we found eight hub proteins with more than 10 disappearing interactions: RUVBL1, RUVBL2, ACTB, KAT5, DMAP1, NFRKB, INO80B and INO80C. RUVBL1 and RUVBL2 are the highly conserved AAA+ chaperone-like ATPases [26] and are involved in various cellular processes, including transcription, DNA repair and RNA modification [27,28]. In the budding yeast *Saccharomyces cerevisiae*, RUVBL1/RUVBL2 homologs (Rvb1/2) interact physically and functionally with cofactors of molecular chaperone Hsp90 that is associated with the formation of NFTs [29,30].

We also found beta-actin (ACTB), which is known as one of the housekeeping genes. However, it was reported that the expression of ACTB is unstable in AD, in real-time quantitative polymerase chain reaction (PCR) [31]. RUVBL1/RUVBL2 and ACTB are present in INO80/SWR1 chromatin-remodeling complex [27,32–34]. Chromatin remodeling controls the epigenetic regulation of gene expression. A recent study showed that the epigenetic suppression of gene expression by increased histone deacetylase 2 prompts cognitive decline [35]. In the present analysis, we showed that the histone acetyltransferase-associated module was damaged in the AD EC region, supporting the proposal that epigenetic inhibition occurs in AD.

 In the RNA polymerase-associated module, five hub proteins were found (RPAP2, MED1, MED12, POLR2G and CTDP1). RNA polymerase II-associated proteins (RPAP1, RPAP2 and RPAP3) provide an interface of RNA polymerase II regulatory complexes (mediator complex and integrator complex), RUVBL1/RUVBL2, and molecular chaperones/scaffolding proteins [36]. MED12 is one of the RNA polymerase II transcriptional mediator subunits, and is implicated in neuronal development and cognitive development [37–40]. The disruption of the RNA polymerase-associated module may contribute to impaired transcription in the AD EC region.

 To detect modules that are affected by the disruption of two identified modules, we searched each late-disrupted module sharing the largest number of disappearing interactions with the histone acetyltransferase-associated module and the RNA polymerase-associated module, respectively. Interestingly, the module sharing disappearing interactions with the histone acetyltransferase-associated module was enriched with a proteasome complex (Figure 9C; modified Fisher’s exact test; *P* = 7.3×10^−63^). One of the major factors of AD is the aggregation of insoluble proteins (e.g., senile plaques and NFTs) and misfolding proteins (e.g., amyloid fibrils). In normal cells, these abnormal proteins are decomposed by protein quality control systems such as the ubiquitin-proteasome system. However, the degradation process of proteins in AD does not work as well as in healthy subjects.

Indeed, an impaired ubiquitin-proteasome system has been observed in AD [41,42]. We found both the deubiquitinating enzyme UCHL5 and subunits of 26S proteasome PSMD7/PSMC4 as hub proteins. Interestingly, UCHL5 in the proteasome was reported to interact with INO80 complex containing RUVBL1/RUVBL2 via NFRKB [43]. In fact, the proteasome-associated module interacts with the histone acetyltransferase-associated module through only UCHL5 (Figure 9C). This suggests that in the AD brains, not only was the ubiquitin-proteasome system impaired by decreased proteasome subunits (PSMD7/PSMC4), but the relationship between proteolysis and transcriptional regulation was also broken down by down-regulated UCHL5.

 The RNA polymerase-associated module shared disappearing interactions with the module related to the transcription factor complex (Figure 9D; modified Fisher’s exact test; *P* = 6.7×10^−46^). We found three hub proteins (ATXN7, TAF10 and SUPT3H) in the transcription factor-associated module. ATXN7 is known as a gene that causes the neurodegenerative disease spinocerebellar ataxia type 7, and it is reported to stabilize microtubules [44]. As we mentioned above, tau protein is associated with the stabilization of microtubules but they cannot work by aberrant phosphorylation in AD. These findings suggest that the decreased expression of ATXN7 promotes the destabilization of microtubules in AD and leads to neuron death.

 We identified hub proteins that have key roles in the mechanisms underlying AD. Our network-based research will be helpful to further filter disease-candidate genes from differentially expressed genes identified by gene expression analyses from the standpoint of network biology.

 In summary, using protein interaction networks (PINs) as a system comprised of multiple network modules, our findings revealed that the modules relevant to the transcriptional regulation are disrupted in the entorhinal cortex region, which affects the ubiquitin-proteasome system.

### Validity for inference of presence/absence of a protein

In our study, we found 76.7% (16,147 genes) of 21,050 genes analyzed were expressed across normal aging and AD tissues. The previous studies reported that 76~86 % of genes were expressed in human brain [4,45,46]. This accordance supports that our threshold to infer presence/absence of a protein is reasonable.

We assumed that a protein was expressed if the corresponding gene was expressed. Schwanhäusser et al. reported the correlation between copies of mRNA and protein was *R* ^2^=0.41 using more than 5,000 genes in mammalian cells [47]. The correlation between copies of mRNA and protein is not high. However, Schwanhäusser also reported that if an impact of transcription, mRNA stability, translation and protein stability on protein abundance is taken into account, predicted protein levels agreed very well with measured protein levels (*R* ^2^=0.85). In this study, we used an expression threshold 200 which is proven to correspond with 3~5 mRNA copies expression per cell experimentally considering an effect of transcription, mRNA stability, translation and protein stability on protein abundance [18]. It thus supports that our threshold is reliable.

 In our study, RNAs in AD and normal aging were extracted from laser-captured postmortem brains and frozen unfixed tissues using different protocols, respectively. Direct comparison between those gene expression values is not appropriate because of including such batch effects. We now identified binarized genes (“expressed” or “unexpressed”) using the threshold, and confirmed that lists of expressed proteins in AD and normal aging are supported by the preceding studies; e.g., in AD, RBAK, RBL1, ZNF268, HOXC4, and HOXB5 genes disappeared along with the Braak stage progression in the HIP region, and in normal aging, OGG1 and MT1G genes appeared along with aging in the HIP region. In AD, transcriptional and tumor suppressor responses activates along with AD progression, and RBAK, RBL1, ZNF268, HOXC4, and HOXB5 genes are known as transcription factors increasing their expressions along with NFT accumulation [48]. In normal aging, reactive oxygen species is produced with age, and the major oxidation product 8-oxoguanine levels increases after 70 years old. To respond the stress, OGG1 gene is considered to over-express with normal aging [49]. MT1G gene is also considered to over-express in aged hippocampus to respond the oxidative stress [49]. Therefore, a list of expressed proteins is reasonable in AD and normal aging, respectively.

## Conclusions

We have shown genome-wide changes of PINs in AD at the following three levels: (1) individual proteins, (2) pairs of known interacting proteins, and (3) sets of proteins called modules. We observed that expressed PINs in the AD EC region lost as many expressed interactions as those of randomized networks. In contrast, expressed PINs in the other brain regions were significantly suppressed, regardless of the AD or normal-aging status of the brain. These results indicate that the EC region, one of the brain regions affected at the early stage in AD, was disrupted at the network level. We also identified AD-disrupted modules (early-disrupted type and late-disrupted type) as disease modules. Interestingly, the number of late-disrupted type modules was greater than that of early-disrupted types across all brain regions, and the number of late-disrupted types in the EC was much greater than that in the HIP and SFG, indicating that with the progression of AD, PINs in the EC rapidly collapse at the module level. Among the late-disrupted modules in the EC region, we found the histone acetyltransferase-associated module and the RNA polymerase-associated module where many expressed interactions disappear with AD progression. We also found each module affected by the disruption of the histone acetyltransferase-associated module and the RNA polymerase-associated module (the proteasome-associated module and the transcription factor-associated module, respectively). Our detailed observations also exposed some hub proteins that contributed to the disruption of the modules. Of these hub proteins, UCHL5 in the proteasome-associated module interacted with the histone acetyltransferase-associated module, suggesting that UCHL5 causes a rupture between epigenetic transcriptional regulation and protein degeneration in AD. Our findings provide the new insight that in AD, the relationship between transcriptional regulation and the ubiquitin-proteasome system is collapsed via the down-regulation of UCHL5.

## Materials and Methods

### The human protein interaction network (PIN)

The human protein interaction dataset was retrieved from the BioGRID (http://thebiogrid.org/; Release 3.1.84) [16,17]. Self-interactions were removed, and the rest of protein interactions were extracted as the human protein interaction network. The human protein interaction network comprises 8,765 proteins and 35,819 interactions.

### Gene expression datasets of postmortem brains of AD subjects and normally aging subjects

A gene expression dataset of the postmortem brains of 48 AD subjects for Braak pathological stages (I‒VI) was retrieved from the U.S. National Center for Biotechnology Information (NCBI) Gene Expression Omnibus (GEO) (http://www.ncbi.nlm.nih.gov/geo/) (GSE5281) [50,51]. The mean postmortem interval (PMI) was 2.5 h. Postmortem brains were laser-captured in six brain regions: entorhinal cortex (EC), hippocampus (HIP), posterior cingulate cortex (PC), superior frontal gyrus (SFG), middle temporal gyrus (MTG) and primary visual cortex (VCX) regions. The Affymetrix U133 Plus 2.0 Array (Affymetrix, Santa Clara, CA) was used for the measurement of gene expression.

 A gene expression dataset of postmortem brains of 55 cognitively intact subjects aged 20 to 99 years was also retrieved from the NCBI GEO (http://www.ncbi.nlm.nih.gov/geo/) [52,53] (GSE11882) [3]. Frozen unfixed tissue was categorized into four brain regions: EC, HIP, SFG, and postcentral gyrus (PCG) regions. The Affymetrix U133 Plus 2.0 Array was used for the measurement of gene expression. We used only the brains of subjects aged 60–99 years as examples of normal aging.

 The gene expression datasets were quality controlled, and we used those in the EC, HIP, and SFG regions that had both a gene expression dataset from postmortem AD brains and a gene expression dataset from cognitively intact brains: 22 AD brains and 18 normal brains in the EC region, 23 AD and 25 normal brains in the HIP region, and 30 AD and 26 normal brains in the SFG.

### Gene-expression data processing in each AD progression stage or in each age group

Gene expression datasets were normalized using the MAS 5.0 algorithm (Affymetrix) to obtain normalized absolute values of gene expressions in each array because they were compared with the absolute threshold based on the previous studies. For each probe set, the average expression values were calculated using the samples marked as “present” by the detection call algorithm (Affymetrix). To reduce as much as possible batch effects, we not only normalized gene expression levels but also used only “Present” call probe sets. We considered that a gene is expressed if the average expression values exceeded 200 [16,17]. We assessed the robustness of our results/conclusions using the different expression threshold (expression levels >150 and > 250) (Figures S5-S8). When a gene had plural probe sets, we adopted the probe set showing the highest variance.

### Identification of expressed protein interaction networks in each AD progression stage or in each age group

We assumed that expressed genes were transcribed to mRNAs, and that mRNAs were translated to proteins. That is, a protein was hypothesized to be expressed if the corresponding gene was expressed. Thus, an expressed protein interaction was defined as a protein interaction whose constituent proteins were expressed at the same time. Expressed protein interactions were also identified in each brain region in each AD progression stage or each age group, and then assembled into expressed PINs in each brain region and in each Braak stage.

### Randomized networks and comparison with an observed value by Z-score

To construct randomized networks of an expressed PIN, we shuffled labels ("expressed" or "unexpressed") assigned to each interaction in all protein interactions without self-interactions retrieved from the BioGRID, and constructed randomized networks from interactions with "expressed" labels (Figure S9). We obtained randomized networks having the same number of interactions as the expressed PIN. We prepared randomized networks for each age group and each AD progression stage, and calculated the ratios of the number of appearing and disappearing interactions to the number of interactions in these randomized networks. This procedure was repeated 1,000 times. We could shuffle labels assigned to each “protein” in all proteins retrieved from the BioGRID, however we did not. If we shuffle labels assigned to each “protein”, we expected that the number of interactions of the randomized network should smaller than that of an original expressed PIN because proteins with a low connection degree tend to be selected due to scale-free property in connection degree. This method would cause low expected values in ratios of appearing/disappearing interactions. We thus did not adopt this randomization procedure. To construct a randomized network keeping the number of interactions, we also could change interacting partners, however we did not. The degree distribution was expected to be kept, however many randomized interactions could not be found in the original interaction set. As mentioned above, appearing/disappearing interactions are defined by whether the interaction includes in interactions selected from the original interaction set. If a lot of interactions were not included in the original interaction set, expected values would be low. We thus did neither adopt this randomization procedure.

 To determine whether the ratios of appearing and disappearing interactions for each age group or each AD progression stage in each brain region were significant, we evaluated the Z-score for statistical significance. The Z-score is defined as follows:


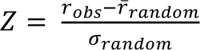
(1)

where *r*_*obs*_ indicates the ratio of appearing or disappearing interactions in an expressed PIN, *r̄_random_* indicates the mean of ratios calculated from 1,000 randomized network sets along with the age group or AD progression stage, and *σ*_*random*_ is the standard deviation of *r*_*random*_.

### Module detection

Modules were detected in each expressed PIN using the Infomap algorithm [20,21]. The infomap algorithm seeks to minimize the description length of a random walker on a network by assigning nodes to modules.  The algorithm uses the map equation to measure the description length and identifies modules in which the random walker tends to stay for a long time. The map equation takes low values for solutions in which a random walker spends long time in (small) modules with infrequent module transitions. For a given network, minimizing the map equation over all possible partitions both gives the optimal assignments of nodes into modules and the optimal number of modules. We set the number of trials to divide a network to 1,000 times. To examine the precision of module detection, we also used the Louvain method [54], the Fast greedy algorithm [55] and the Markov cluster algorithm (MCL) [56]. The MCL’s inflation option was set to 4.0. In our study, we used only modules composed of three or more expressed proteins through all algorithms.

### Auto-correlation of proteins (*C_N_*), interactions (*C_L_*), and cellular functions (*C_GO_*)

To quantify how frequently a module changes its constituent proteins, interactions and cellular functions with aging and with AD progression, we defined the auto-correlation of proteins (*C*_*N*_), interactions (*C*_*L*_) and cellular functions (*C*_*GO*_) as follows [57]:


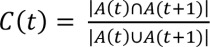
(2)

where A(t) is a set of proteins (*C*_*N*_), interactions (*C*_*L*_) and cellular functions (*C*_*GO*_) in a module at time *t* (i.e., an age group or an AD progression stage), 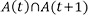 is the number of the common proteins (*C_N_*), interactions (*C_L_*) and cellular functions (*C*_GO_) between a module at time *t* and a module at time *t*+1, and 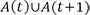 is the number of proteins (*C_N_*), interactions (*C_L_*) and cellular functions (*C* *_GO_*) in the union between a module at time *t* and a module at time *t*+1. We retrieved cellular functions from the “biological process” of the Gene Ontology Annotation (GOA) [58].

### Inherited module lineage and disrupted inherited-module lineage

If a module pair exhibits the highest *C*_*L*_ in two consecutive stages and their *C*_*L*_ and *C*_*GO*_ exceeded 0.5 (i.e., over half), we assumed that the modules were inherited. For example, in Figure 5, there are two and three modules at time *t* and *t*+1, respectively. To seek a module at time *t*+1 inheriting a module at time *t*, we made a bipartite graph (Figure 5A). The number of links is six. We computed *C*_*L*_ between a module at time *t* and a module at time *t*+1, and identified module pairs with the highest *C*_*L*_ for both module at time *t* and *t*+1 (e.g. a red module in time *t* and a green module in time *t*+1). If their *C*_*L*_ and *C*_*GO*_ exceeded 0.5, a module at time *t*+1 inherits from the module at time *t*. That is, we considered that a red module at time *t* and a green module at time *t*+1, a blue module at time *t* and a purple module at time *t*+1 are same in Figure 5B. We repeated these procedures. If their modules were inherited from the earliest age group or earliest AD progression stage (60–69 y/o or Braak stage I) to the latest age group or AD progression stage (90–99 y/o or Braak stage IV in the EC region or Braak stage VI in the HIP and the SFG regions), we called these modules “inherited-module lineages,” and called the other modules “disrupted inherited-module lineages.”

### Enrichment analysis of module function

We performed enrichment analyses to assign functions to a module using the following procedures. First, we assigned the GOA common to both proteins constituting an interaction to the interaction. GOAs were simplified by manual curation. We repeated this procedure for all interactions. Second, we considered subset S1 and subset S2. Each S1 is an interaction set in a module, and each S2 is an interaction set with a function. The significance of the overlap between S1 and S2 was evaluated by determining the hypergeometric distribution and fold enrichment ratio (FER) as follows:


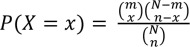
(3)


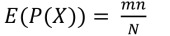
(4)


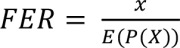
(5)

where *x* is the number of interactions that overlapped between S1 and S2, and *m* and *n* are the numbers of interactions in S1 and S2, respectively. *N* is the total number of interactions with GO functions. If the probability by hypergeometric distribution was less than 0.05 and the FER was greater than 2, we assigned the GOA to the module.

## Supporting Information

Figure S1 **The correlation of the gene expression levels of proteins that appear/disappear with aging.** A boxplot represents the gene expression levels of proteins that appear/disappear in each aging group (60‒69, 70‒79, 80‒89, 90‒99 years old). A red line indicates expression level 200 as threshold. The gene expression levels significantly correlated with aging.
(TIFF)

Figure S2 **The correlation of the gene expression levels of proteins that appear/disappear with AD progression.** A boxplot represents the gene expression levels of proteins that appear/disappear in each AD progression stages (Braak stage I, II, III/IV, V/VI). A red line indicates expression level 200 as threshold. The gene expression levels significantly correlated with AD progression.
(TIFF)

Figure S3 **Ratio of inherited module lineages using different thresholds.** The figure shows the ratio of inherited module lineages to the total number of module lineages using different *C*_*L*_ and *C*_*GO*_ (i.e. 0.3, 0.4, 0.5 (default), 0.6, 0.7). The ratios of inherited module lineages in AD were lower than those in normal aging through all brain regions.
(TIFF)

Figure S4 **The correlation between module size and a kind of module.** Module size is interpreted as the number of proteins in the union among the inherited modules. A boxplot represents the number of proteins in the union among the inherited modules. Multiple comparison was perfomed by Kruskal-Wallis test. As a result, module sizes of inherited module lineage were significantly ~2.2-fold higher than them of appearing/disappearing module lineages. On the other hand, we did not find differences of module sizes between appearing and disappearing module lineages.
(TIFF)

Figure S5 **The number of expressed proteins and interactions in expressed PINs for two different thresholds.** When a gene is expressed, if the average expression value exceeded 150, the boxplot represents the numbers of (A) expressed proteins or (B) expressed interactions for normal age groups (60‒69, 70‒79, 80‒89, 90‒99 years old) and AD progression stages (Braak stage I, II, III/IV, V/VI). When the threshold was 250, (C) and (D) show the numbers of expressed proteins and expressed interactions, respectively. As with the main text (threshold 200), the numbers of expressed proteins and interactions in the AD EC and HIP were significantly lower than those in the normal aging groups (Wilcoxon test; P < 0.05, respectively).
(TIFF)

Figure S6 **Ratio of appearing and disappearing interactions for two different thresholds.** Red and blue plots indicate ratios of newly appearing and disappearing protein interactions, respectively. Boxplots indicate ratios of appearing and disappearing protein interactions from 1,000 corresponding randomized networks in each brain region in normal aging and AD. Values below the boxplots show the Z-scores between the ratio and the ratios of the 1,000 randomized networks. The ratio of the number of disappearing interactions in the AD EC region showed no significant difference from those of the 1,000 randomized networks for two different thresholds, 150 and 250, at which a gene is expressed (Z-score = −1.24 and Z-score = −0.614, respectively).
(TIFF)

Figure S7 **Auto-correlations of proteins, interactions, and functions for inherited modules using the threshold 150.** Probability density distributions of (A) auto-correlations of proteins, (B) interactions, and (C) cellular functions of a consecutive module pair. Orange and green curves indicate normal aging and AD, respectively. P-values were calculated from the Wilcoxon test. Auto-correlations in AD were significantly lower than those in normal aging through all brain regions.
(TIFF)

Figure S8 **Auto-correlations of proteins, interactions, and functions for inherited modules using the threshold 250.** Probability density distributions of (A) auto-correlations of proteins, (B) interactions, and (C) cellular functions of a consecutive module pair. Orange and green curves indicate normal aging and AD, respectively. P-values were calculated from the Wilcoxon test. Auto-correlations in AD were significantly lower than those in normal aging through all brain regions.
(TIFF)

Figure S9 **A scheme for constructing a randomized network.** To construct randomized networks of an expressed PIN, we shuffled labels ("expressed" or "unexpressed") assigned to each interaction in all protein interactions without self-interactions retrieved from the BioGRID, and made randomized networks from interactions with "expressed" labels. We obtained randomized networks having the same number of interactions as the expressed PIN.
(TIFF)

Table S1 **Q-modularity and the number of proteins included in a module.** To evaluate the quality of divisions of a network, we compared Q-modularity among four algorithms. The Q-modularity of a network with strong module structure usually falls in the range between 0.3 and 0.7. Infomap, Louvain and Fast greedy algorithms had more than 0.3 Q-modularity, and suited our expressed PINs to divide into modules. We also examined the number of proteins included in a module. Consequently, each maximum module by the Louvain and Fast greedy algorithms included more than half of all proteins in the PIN. The maximum module obtained with the Infomap algorithm included only 22.7% in all proteins in the PIN. The Infomap algorithm had high Q-modularity and finely divided the PINs into modules compared to the other methods. We therefore used the Infomap algorithm.
(PDF)

Table S2 **Summary for module sizes in inherited module lineages and appearing/disappearing module lineages.** Module size is interpreted as the number of proteins in the union among the inherited modules. Medians of module sizes were shown. The module sizes of inheireted module lienages per them of appearing/disappearing module lineages were also shown.
(PDF)

Table S3 **The list of AD-disrupted modules.** The “Enriched GO annotation” column indicates significant cellular functions by an enrichment analysis. The “Gene symbol” column indicates genes expressed once in stages. In cases of early- and late-disrupted type modules, “Gene symbol” shows genes in AD modules that corresponded to modules in normal aging.
(PDF)

## References

[1] Braak H, Braak E (1991) Neuropathological staging of Alzheimer-related changes. Acta Neuropathol 82: 239–259. doi:10.1007/BF00308809. PubMed: 1759558. 1759558

[2] Haroutunian V, Katsel P, Schmeidler J (2009) Transcriptional vulnerability of brain regions in Alzheimer's disease and dementia. Neurobiol Aging 30: 561-573. doi:10.1016/j.neurobiolaging.2007.07.021. PubMed: 17845826. 17845826 PMC2746014

[3] Berchtold NC, Cribbs DH, Coleman PD, Rogers J, Head E et al. (2008) Gene expression changes in the course of normal brain aging are sexually dimorphic. Proc Natl Acad Sci U_S_A 105: 15605-15610. doi:10.1073/pnas.0806883105. PubMed: 18832152. 18832152 PMC2563070

[4] Kang HJ, Kawasawa YI, Cheng F, Zhu Y, Xu X et al. (2011) Spatio-temporal transcriptome of the human brain. Nature 478: 483-489. doi:10.1038/nature10523. PubMed: 22031440. 22031440 PMC3566780

[5] Chen L, Liu R, Liu ZP, Li M, Aihara K (2012) Detecting early-warning signals for sudden deterioration of complex diseases by dynamical network biomarkers. Sci Rep 2: 342 PubMed: 22461973. 22461973 10.1038/srep00342 PMC3314989

[6] Barabási AL, Gulbahce N, Loscalzo J (2011) Network medicine: a network-based approach to human disease. Nat Rev Genet 12: 56-68. doi:10.1038/nrg2918. PubMed: 21164525. 21164525 PMC3140052

[7] Miller JA, Oldham MC, Geschwind DH (2008) A systems level analysis of transcriptional changes in Alzheimer's disease and normal aging. J Neurosci 28: 1410-1420. doi:10.1523/JNEUROSCI.4098-07.2008. PubMed: 18256261. 18256261 PMC2902235

[8] Ray M, Zhang W (2010) Analysis of Alzheimer's disease severity across brain regions by topological analysis of gene co-expression networks. BMC. Syst Biol 4: 136. 10.1186/1752-0509-4-136 PMC2976747 20925940

[9] Liu ZP, Wang Y, Zhang XS, Chen L (2010) Identifying dysfunctional crosstalk of pathways in various regions of Alzheimer's disease brains. BMC. Syst Biol 4 Suppl 2: S11. 10.1186/1752-0509-4-S2-S11 PMC2982685 20840725

[10] Squire LR (1992) Memory and the hippocampus: a synthesis from findings with rats, monkeys, and humans. Psychol Rev 99: 195-231. doi:10.1037/0033-295X.99.2.195. PubMed: 1594723. 1594723

[11] Suthana N, Haneef Z, Stern J, Mukamel R, Behnke E et al. (2012) Memory enhancement and deep-brain stimulation of the entorhinal area. N Engl J Med 366: 502-510. doi:10.1056/NEJMoa1107212. PubMed: 22316444. 22316444 PMC3447081

[12] du Boisgueheneuc F, Levy R, Volle E, Seassau M, Duffau H et al. (2006) Functions of the left superior frontal gyrus in humans: a lesion study. Brain 129: 3315-3328. doi:10.1093/brain/awl244. PubMed: 16984899. 16984899

[13] Evans DA, Funkenstein HH, Albert MS, Scherr PA, Cook NR et al. (1989) Prevalence of Alzheimer's disease in a community population of older persons. Higher than previously reported. JAMA 262: 2551-2556. doi:10.1001/jama.1989.03430180093036. PubMed: 2810583. 2810583

[14] Hebert LE, Scherr PA, Bienias JL, Bennett DA, Evans DA (2003) Alzheimer disease in the US population: prevalence estimates using the 2000 census. Arch Neurol 60: 1119-1122. doi:10.1001/archneur.60.8.1119. PubMed: 12925369. 12925369

[15] Brookmeyer R, Gray S, Kawas C (1998) Projections of Alzheimer's disease in the United States and the public health impact of delaying disease onset. Am J Public Health 88: 1337-1342. doi:10.2105/AJPH.88.9.1337. PubMed: 9736873. 9736873 PMC1509089

[16] Stark C, Breitkreutz BJ, Reguly T, Boucher L, Breitkreutz A et al. (2006) BioGRID: a general repository for interaction datasets. Nucleic Acids Res 34: D535-D539. doi:10.1093/nar/gkj109. PubMed: 16381927. 16381927 PMC1347471

[17] Stark C, Breitkreutz BJ, Chatr-Aryamontri A, Boucher L, Oughtred R et al. (2011) The BioGRID Interaction Database: 2011 update. Nucleic Acids Res 39: D698-D704. doi:10.1093/nar/gkq1116. PubMed: 21071413. 21071413 PMC3013707

[18] Su AI, Cooke MP, Ching KA, Hakak Y, Walker JR et al. (2002) Large-scale analysis of the human and mouse transcriptomes. Proc Natl Acad Sci U_S_A 99: 4465-4470. doi:10.1073/pnas.012025199. PubMed: 11904358. 11904358 PMC123671

[19] Bossi A, Lehner B (2009) Tissue specificity and the human protein interaction network. Mol Syst Biol 5: 260 PubMed: 19357639. 19357639 10.1038/msb.2009.17 PMC2683721

[20] Rosvall M, Bergstrom CT (2008) Maps of random walks on complex networks reveal community structure. Proc Natl Acad Sci U S A 105: 1118-1123. doi:10.1073/pnas.0706851105. PubMed: 18216267. 18216267 PMC2234100

[21] Rosvall M, Axelsson D, Bergstrom CT (2008). The map equation. arXiv:0906.1405v2.

[22] Lancichinetti A, Fortunato S (2009) Community detection algorithms: a comparative analysis. Phys Rev E 80: 056117. doi:10.1103/PhysRevE.80.056117. PubMed: 20365053. 20365053

[23] Newman ME (2006) Modularity and community structure in networks. Proc Natl Acad Sci U_S_A 103: 8577-8582. doi:10.1073/pnas.0601602103. PubMed: 16723398. 16723398 PMC1482622

[24] Huang DW, Sherman BT, Tan Q, Collins JR, Alvord WG et al. (2007) The DAVID Gene Functional Classification Tool: a novel biological module-centric algorithm to functionally analyze large gene lists. Genome Biol 8: R183. doi:10.1186/gb-2007-8-9-r183. PubMed: 17784955. 17784955 PMC2375021

[25] Huang DW, Sherman BT, Lempicki RA (2009) Systematic and integrative analysis of large gene lists using DAVID bioinformatics resources. Nat Protoc 4: 44-57. PubMed: 19131956. 19131956 10.1038/nprot.2008.211

[26] Doyon Y, Côté J (2004) The highly conserved and multifunctional NuA4 HAT complex. Curr Opin Genet Dev 14: 147-154. doi:10.1016/j.gde.2004.02.009. PubMed: 15196461. 15196461

[27] Ikura T, Ogryzko VV, Grigoriev M, Groisman R, Wang J et al. (2000) Involvement of the TIP60 histone acetylase complex in DNA repair and apoptosis. Cell 102: 463-473. doi:10.1016/S0092-8674(00)00051-9. PubMed: 10966108. 10966108

[28] Jha S, Dutta A (2009) RVB1/RVB2: running rings around molecular biology. Mol Cell 34: 521-533. doi:10.1016/j.molcel.2009.05.016. PubMed: 19524533. 19524533 PMC2733251

[29] Zhao R, Davey M, Hsu YC, Kaplanek P, Tong A et al. (2005) Navigating the chaperone network: an integrative map of physical and genetic interactions mediated by the hsp90 chaperone. Cell 120: 715-727. doi:10.1016/j.cell.2004.12.024. PubMed: 15766533. 15766533

[30] Salminen A, Ojala J, Kaarniranta K, Hiltunen M, Soininen H (2011) Hsp90 regulates tau pathology through co-chaperone complexes in Alzheimer's disease. Prog Neurobiol 93: 99-110. doi:10.1016/j.pneurobio.2010.10.006. PubMed: 21056617. 21056617

[31] Leduc V, Legault V, Dea D, Poirier J (2011) Normalization of gene expression using SYBR green qPCR: a case for paraoxonase 1 and 2 in Alzheimer's disease brains. J Neurosci Methods 200: 14-19. doi:10.1016/j.jneumeth.2011.05.026. PubMed: 21672555. 21672555

[32] Cai Y, Jin J, Tomomori-Sato C, Sato S, Sorokina I et al. (2003) Identification of new subunits of the multiprotein mammalian TRRAP/TIP60-containing histone acetyltransferase complex. J Biol Chem 278: 42733-42736. doi:10.1074/jbc.C300389200. PubMed: 12963728. 12963728

[33] Jin J, Cai Y, Yao T, Gottschalk AJ, Florens L et al. (2005) A mammalian chromatin remodeling complex with similarities to the yeast INO80 complex. J Biol Chem 280: 41207-41212. doi:10.1074/jbc.M509128200. PubMed: 16230350. 16230350

[34] Morrison AJ, Shen X (2009) Chromatin remodelling beyond transcription: the INO80 and SWR1 complexes. Nat Rev Mol Cell Biol 10: 373-384. doi:10.1038/nrm2693. PubMed: 19424290. 19424290 PMC6103619

[35] Gräff J, Rei D, Guan JS, Wang WY, Seo J et al. (2012) An epigenetic blockade of cognitive functions in the neurodegenerating brain. Nature 483: 222-226. doi:10.1038/nature10849. PubMed: 22388814. 22388814 PMC3498952

[36] Jeronimo C, Forget D, Bouchard A, Li Q, Chua G et al. (2007) Systematic analysis of the protein interaction network for the human transcription machinery reveals the identity of the 7SK capping enzyme. Mol Cell 27: 262-274. doi:10.1016/j.molcel.2007.06.027. PubMed: 17643375. 17643375 PMC4498903

[37] Risheg H, Graham JM, Clark RD, Rogers RC, Opitz JM et al. (2007) A recurrent mutation in MED12 leading to R961W causes Opitz-Kaveggia syndrome. Nat Genet 39: 451-453. doi:10.1038/ng1992. PubMed: 17334363. 17334363

[38] Rump P, Niessen RC, Verbruggen KT, Brouwer OF, de Raad M et al. (2011) A novel mutation in MED12 causes FG syndrome (Opitz-Kaveggia syndrome). Clin Genet 79: 183-188. doi:10.1111/j.1399-0004.2010.01449.x. PubMed: 20507344. 20507344

[39] Ding N, Zhou H, Esteve PO, Chin HG, Kim S et al. (2008) Mediator links epigenetic silencing of neuronal gene expression with x-linked mental retardation. Mol Cell 31: 347-359. doi:10.1016/j.molcel.2008.05.023. PubMed: 18691967. 18691967 PMC2583939

[40] Wang X, Yang N, Uno E, Roeder RG, Guo S (2006) A subunit of the mediator complex regulates vertebrate neuronal development. Proc Natl Acad Sci U_S_A 103: 17284-17289. doi:10.1073/pnas.0605414103. PubMed: 17088561. 17088561 PMC1859923

[41] Keller JN, Hanni KB, Markesbery WR (2000) Impaired proteasome function in Alzheimer's disease. J Neurochem 75: 436-439. PubMed: 10854289. 10854289 10.1046/j.1471-4159.2000.0750436.x

[42] Lam YA, Pickart CM, Alban A, Landon M, Jamieson C et al. (2000) Inhibition of the ubiquitin-proteasome system in Alzheimer's disease. Proc Natl Acad Sci U_S_A 97: 9902-9906. doi:10.1073/pnas.170173897. PubMed: 10944193. 10944193 PMC27620

[43] Yao T, Song L, Jin J, Cai Y, Takahashi H et al. (2008) Distinct modes of regulation of the Uch37 deubiquitinating enzyme in the proteasome and in the Ino80 chromatin-remodeling complex. Mol Cell 31: 909-917. doi:10.1016/j.molcel.2008.08.027. PubMed: 18922472. 18922472 PMC2577292

[44] Nakamura Y, Tagawa K, Oka T, Sasabe T, Ito H et al. (2012) Ataxin-7 associates with microtubules and stabilizes the cytoskeletal network. Hum Mol Genet 21: 1099-1110. doi:10.1093/hmg/ddr539. PubMed: 22100762. 22100762 PMC3277310

[45] Johnson MB, Kawasawa YI, Mason CE, Krsnik Z, Coppola G et al. (2009) Functional and evolutionary insights into human brain development through global transcriptome analysis. Neuron 62: 494-509. doi:10.1016/j.neuron.2009.03.027. PubMed: 19477152. 19477152 PMC2739738

[46] Hawrylycz MJ, Lein ES, Guillozet-Bongaarts AL, Shen EH, Ng L et al. (2012) An anatomically comprehensive atlas of the adult human brain transcriptome. Nature 489: 391-399. doi:10.1038/nature11405. PubMed: 22996553. 22996553 PMC4243026

[47] Schwanhäusser B, Busse D, Li N, Dittmar G, Schuchhardt J et al. (2011) Global quantification of mammalian gene expression control. Nature 473: 337-342. doi:10.1038/nature10098. PubMed: 21593866. 21593866

[48] Blalock EM, Geddes JW, Chen KC, Porter NM, Markesbery WR et al. (2004) Incipient Alzheimer's disease: microarray correlation analyses reveal major transcriptional and tumor suppressor responses. Proc Natl Acad Sci U S A 101: 2173-2178. doi:10.1073/pnas.0308512100. PubMed: 14769913. 14769913 PMC357071

[49] Lu T, Pan Y, Kao SY, Li C, Kohane I et al. (2004) Gene regulation and DNA damage in the ageing human brain. Nature 429: 883-891. doi:10.1038/nature02661. PubMed: 15190254. 15190254

[50] Liang WS, Dunckley T, Beach TG, Grover A, Mastroeni D et al. (2007) Gene expression profiles in anatomically and functionally distinct regions of the normal aged human brain. Physiol Genomics 28: 311-322. PubMed: 17077275. 17077275 10.1152/physiolgenomics.00208.2006 PMC2259385

[51] Liang WS, Dunckley T, Beach TG, Grover A, Mastroeni D et al. (2008) Altered neuronal gene expression in brain regions differentially affected by Alzheimer's disease: a reference data set. Physiol Genomics 33: 240-256. doi:10.1152/physiolgenomics.00242.2007. PubMed: 18270320. 18270320 PMC2826117

[52] Barrett T, Troup DB, Wilhite SE, Ledoux P, Rudnev D et al. (2007) NCBI GEO: mining tens of millions of expression profiles--database and tools update. Nucleic Acids Res 35: D760-D765. doi:10.1093/nar/gkl887. PubMed: 17099226. 17099226 PMC1669752

[53] Barrett T, Troup DB, Wilhite SE, Ledoux P, Rudnev D et al. (2009) NCBI GEO: archive for high-throughput functional genomic data. Nucleic Acids Res 37: D885-D890. doi:10.1093/nar/gkn764. PubMed: 18940857. 18940857 PMC2686538

[54] Blondel VD, Guillaume JL, Lambiotte R, Lefebvre E (2008) Fast unfolding of communities in large networks. J Stat Mech P10008. doi:10.1088/1742-5468/2008/10/P10008.

[55] Clauset A, Newman MEJ, Moore C (2004) Finding community structure in very large networks. Phys Rev E 70: 066111. doi:10.1103/PhysRevE.70.066111. PubMed: 15697438. 15697438

[56] Enright AJ, Van Dongen S, Ouzounis CA (2002) An efficient algorithm for large-scale detection of protein families. Nucleic Acids Res 30: 1575-1584. doi:10.1093/nar/30.7.1575. PubMed: 11917018. 11917018 PMC101833

[57] Palla G, Barabási AL, Vicsek T (2007) Quantifying social group evolution. Nature 446: 664-667. doi:10.1038/nature05670. PubMed: 17410175. 17410175

[58] Ashburner M, Ball CA, Blake JA, Botstein D, Butler H et al. (2000) Gene ontology: tool for the unification of biology. The Gene Ontology Consortium. Nat Genet 25: 25-29. doi:10.1038/75556. PubMed: 10802651. 10802651 PMC3037419

