# Supplementary figures and images for: Identification of Unstable Network Modules Reveals Disease Modules Associated with the Progression of Alzheimer’s Disease
Source: PLoS One. 2013 Nov 15;8(11):e76162. doi: 10.1371/journal.pone.0076162 (PMC3858171; doi:10.1371/journal.pone.0076162)

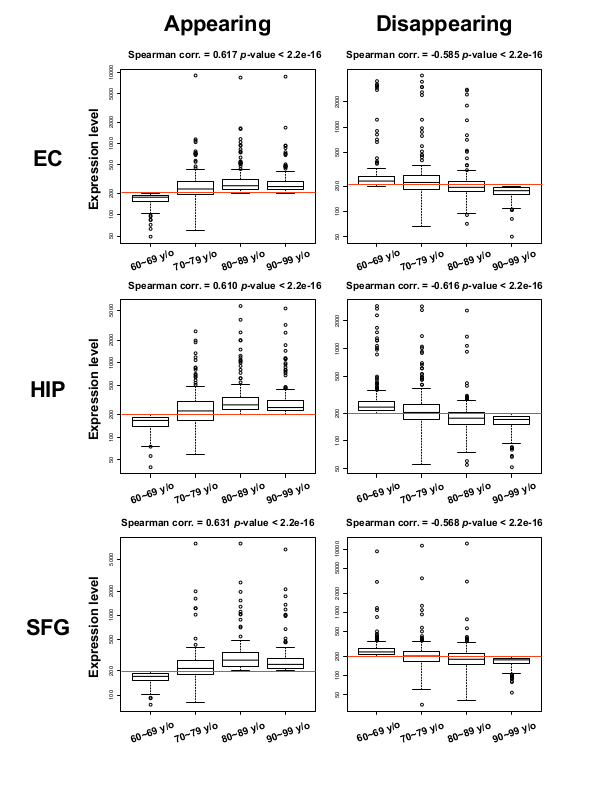

Supplement: Figure S1 — The correlation of the gene expression levels of proteins that appear/disappear with aging. A boxplot represents the gene expression levels of proteins that appear/disappear in each aging group (60‒69, 70‒79, 80‒89, 90‒99 years old). A red line indicates expression level 200 as threshold. The gene expression levels significantly correlated with aging. (TIFF) [file pone.0076162.s001.tiff]

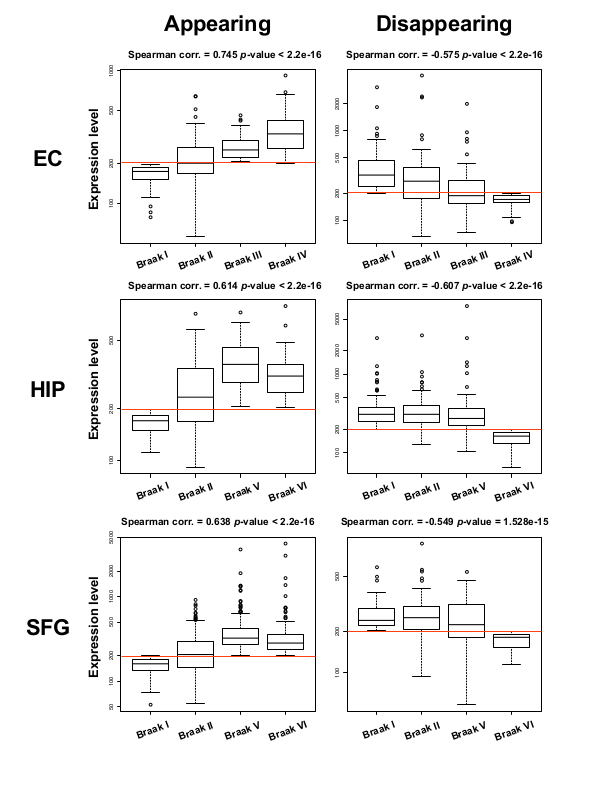

Supplement: Figure S2 — The correlation of the gene expression levels of proteins that appear/disappear with AD progression. A boxplot represents the gene expression levels of proteins that appear/disappear in each AD progression stages (Braak stage I, II, III/IV, V/VI). A red line indicates expression level 200 as threshold. The gene expression levels significantly correlated with AD progression. (TIFF) [file pone.0076162.s002.tiff]

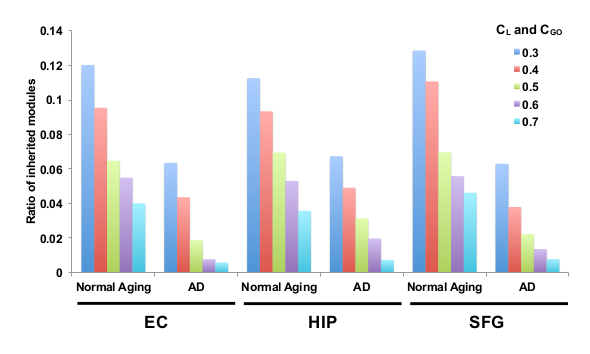

Supplement: Figure S3 — Ratio of inherited module lineages using different thresholds. The figure shows the ratio of inherited module lineages to the total number of module lineages using different C L and C GO (i.e. 0.3, 0.4, 0.5 (default), 0.6, 0.7). The ratios of inherited module lineages in AD were lower than those in normal aging through all brain regions. (TIFF) [file pone.0076162.s003.tiff]

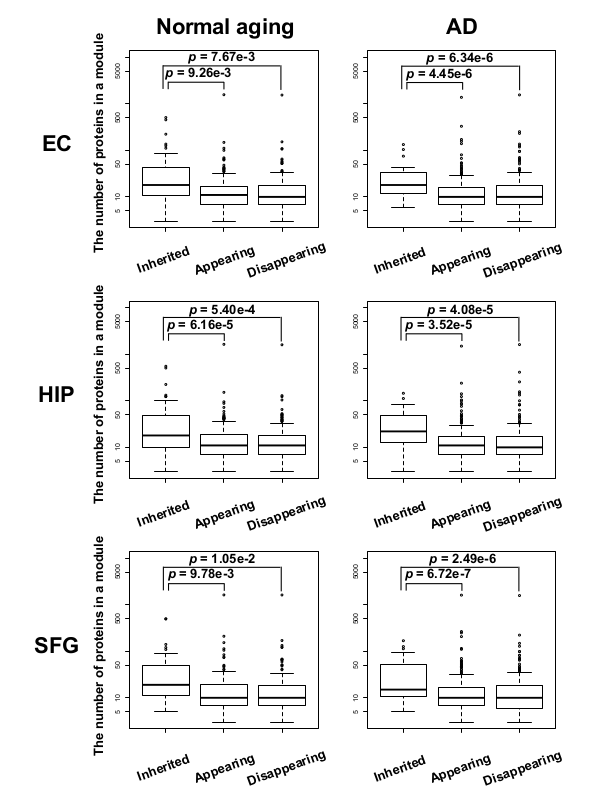

Supplement: Figure S4 — The correlation between module size and a kind of module. Module size is interpreted as the number of proteins in the union among the inherited modules. A boxplot represents the number of proteins in the union among the inherited modules. Multiple comparison was perfomed by Kruskal-Wallis test. As a result, module sizes of inherited module lineage were significantly ~2.2-fold higher than them of appearing/disappearing module lineages. On the other hand, we did not find differences of module sizes between appearing and disappearing module lineages. (TIFF) [file pone.0076162.s004.tiff]

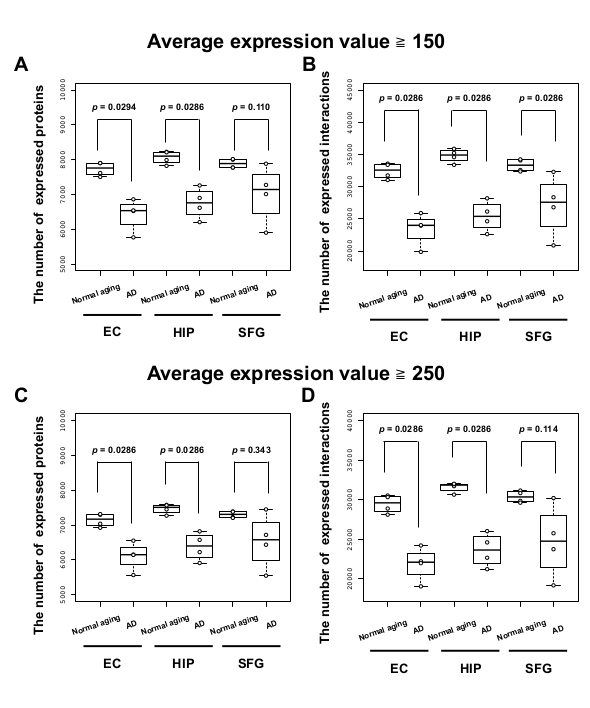

Supplement: Figure S5 — The number of expressed proteins and interactions in expressed PINs for two different thresholds. When a gene is expressed, if the average expression value exceeded 150, the boxplot represents the numbers of (A) expressed proteins or (B) expressed interactions for normal age groups (60‒69, 70‒79, 80‒89, 90‒99 years old) and AD progression stages (Braak stage I, II, III/IV, V/VI). When the threshold was 250, (C) and (D) show the numbers of expressed proteins and expressed interactions, respectively. As with the main text (threshold 200), the numbers of expressed proteins and interactions in the AD EC and HIP were significantly lower than those in the normal aging groups (Wilcoxon test; P < 0.05, respectively). (TIFF) [file pone.0076162.s005.tiff]

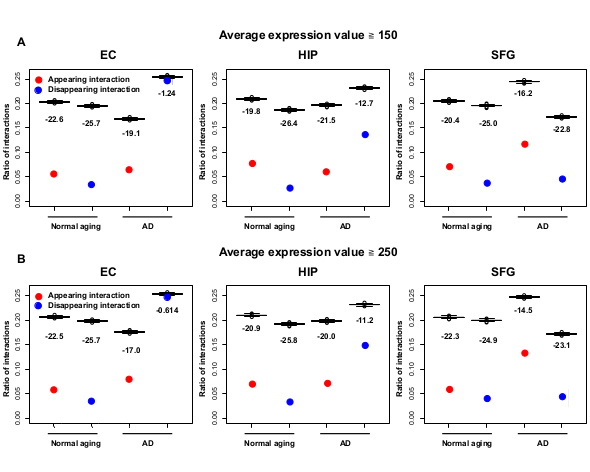

Supplement: Figure S6 — Ratio of appearing and disappearing interactions for two different thresholds. Red and blue plots indicate ratios of newly appearing and disappearing protein interactions, respectively. Boxplots indicate ratios of appearing and disappearing protein interactions from 1,000 corresponding randomized networks in each brain region in normal aging and AD. Values below the boxplots show the Z-scores between the ratio and the ratios of the 1,000 randomized networks. The ratio of the number of disappearing interactions in the AD EC region showed no significant difference from those of the 1,000 randomized networks for two different thresholds, 150 and 250, at which a gene is expressed (Z-score = −1.24 and Z-score = −0.614, respectively). (TIFF) [file pone.0076162.s006.tiff]

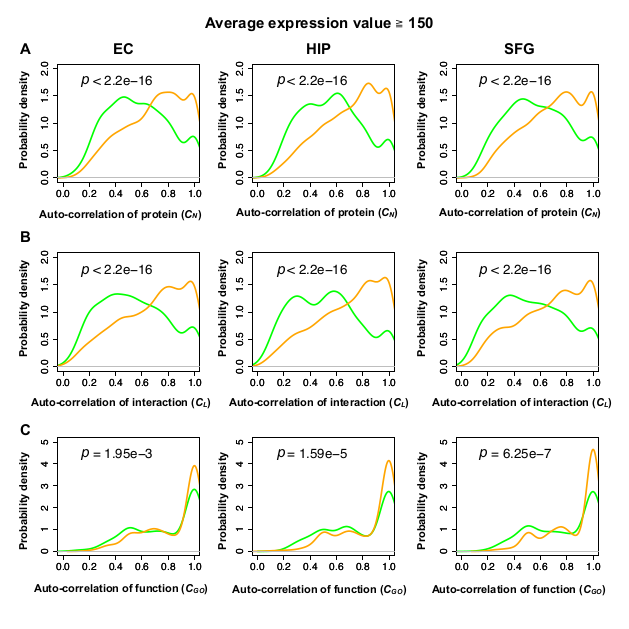

Supplement: Figure S7 — Auto-correlations of proteins, interactions, and functions for inherited modules using the threshold 150. Probability density distributions of (A) auto-correlations of proteins, (B) interactions, and (C) cellular functions of a consecutive module pair. Orange and green curves indicate normal aging and AD, respectively. P-values were calculated from the Wilcoxon test. Auto-correlations in AD were significantly lower than those in normal aging through all brain regions. (TIFF) [file pone.0076162.s007.tiff]

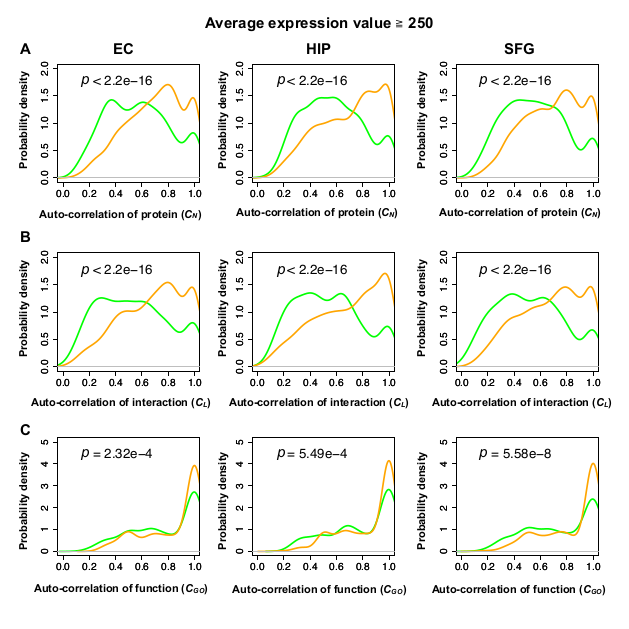

Supplement: Figure S8 — Auto-correlations of proteins, interactions, and functions for inherited modules using the threshold 250. Probability density distributions of (A) auto-correlations of proteins, (B) interactions, and (C) cellular functions of a consecutive module pair. Orange and green curves indicate normal aging and AD, respectively. P-values were calculated from the Wilcoxon test. Auto-correlations in AD were significantly lower than those in normal aging through all brain regions. (TIFF) [file pone.0076162.s008.tiff]

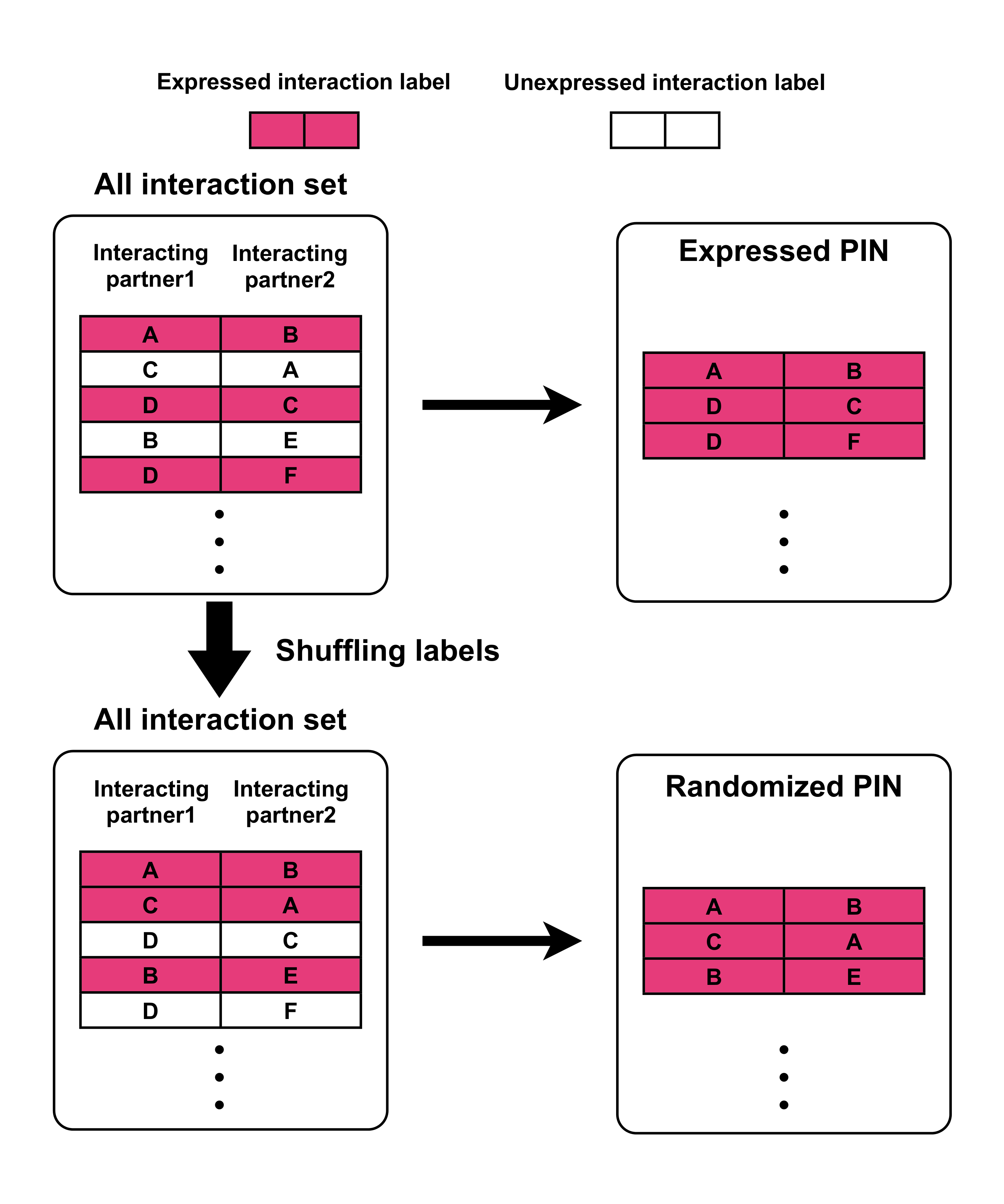

Supplement: Figure S9 — A scheme for constructing a randomized network. To construct randomized networks of an expressed PIN, we shuffled labels ("expressed" or "unexpressed") assigned to each interaction in all protein interactions without self-interactions retrieved from the BioGRID, and made randomized networks from interactions with "expressed" labels. We obtained randomized networks having the same number of interactions as the expressed PIN. (TIFF) [file pone.0076162.s009.tiff]
