# Supplementary material for: Identification of Unstable Network Modules Reveals Disease Modules Associated with the Progression of Alzheimer’s Disease
Source: PLoS One. 2013 Nov 15;8(11):e76162. doi: 10.1371/journal.pone.0076162 (PMC3858171; doi:10.1371/journal.pone.0076162)
Supplement: Table S1 — Q-modularity and the number of proteins included in a module. To evaluate the quality of divisions of a network, we compared Q-modularity among four algorithms. The Q-modularity of a network with strong module structure usually falls in the range between 0.3 and 0.7. Infomap, Louvain and Fast greedy algorithms had more than 0.3 Q-modularity, and suited our expressed PINs to divide into modules. We also examined the number of proteins included in a module. Consequently, each maximum module by the Louvain and Fast greedy algorithms included more than half of all proteins in the PIN. The maximum module obtained with the Infomap algorithm included only 22.7% in all proteins in the PIN. The Infomap algorithm had high Q-modularity and finely divided the PINs into modules compared to the other methods. We therefore used the Infomap algorithm. (PDF) [file pone.0076162.s010.pdf]

	Q-Modularity				The number of proteins included in one module			
	Min.	Median	Mean	Max.	Min.	Median	Mean	Max.
Infomap	0.3902	0.3961	0.3966	0.4097	3	13	40.46	1992
Louvain	0.431	0.4396	0.4411	0.4502	3	212	583.1	5663
Fast greedy	0.3738	0.3832	0.385	0.3987	3	6	281.5	5663
MCL	0.1027	0.1081	0.1091	0.1184	3	3	43.47	5663
