# Supplementary material for: Identification of Unstable Network Modules Reveals Disease Modules Associated with the Progression of Alzheimer’s Disease
Source: PLoS One. 2013 Nov 15;8(11):e76162. doi: 10.1371/journal.pone.0076162 (PMC3858171; doi:10.1371/journal.pone.0076162)
Supplement: Table S2 — Summary for module sizes in inherited module lineages and appearing/disappearing module lineages. Module size is interpreted as the number of proteins in the union among the inherited modules. Medians of module sizes were shown. The module sizes of inheireted module lienages per them of appearing/disappearing module lineages were also shown. (PDF) [file pone.0076162.s011.pdf]

Brain region	Type	Enriched GO annotation	Gene symbol
EC	Common type	DNA replication, Protein catabolic process, Transport	AQR, ARMC7, BTBD9, HACL1, KCTD10, KCTD13, LSM6, MT2A, MYBPC1, POLD2, SRPX, TNFAIP1, USP25, USP28, ZMYND19
EC	Common type	Transport	KIAA1549, MBD5, MYO5B, RAB11A, RAB11FIP1, RAB11FIP2, RAB11FIP3, RAB11FIP4, RAB11FIP5, REPS1
EC	Common type	Cellular component organization, Kinase activity, Localization, Response to stimulus, Transcription	ACD, BMP2K, C1orf63, CDK13, CLK3, CYLC2, DDX3Y, FOXJ3, GNL3L, GRPEL1, KIAA2022, MACROD2, NANOG, PAK1IP1, PCBP1, PCMT1, PLOD1, PLOD2, POT1, PRPS1L1, RASGEF1C, RBM15B, RPS7, RSBN1L, SALL1, SEC16A, TDRD6, TERF1, TERF2IP, TINF2, YTHDF1, YTHDF3, ZC3HAV1L, ZNF281
EC	Common type	DNA repair, Apoptosis, Protein catabolic process, Protein metabolic process, Protein modification process	AGFG1, AKR1C3, ARIH1, ARIH2, BCL10, BFAR, BIRC2, BIRC3, BIRC6, BIRC7, C9orf89, CADPS2, CNOT4, DDX58, DIABLO, DTX1, DTX3L, DZIP3, EIF4E2, EML4, EPS15, EXTL3, FBXL19, FCHO2, GPR108, HLTF, HPCA, HTRA2, INF2, ISG15, KIAA1107, KIAA1797, LNX2, LOC147791, LRSAM1, LTN1, MAGEL2, MAP3K1, MARCH5, MARCH7, MC1R, MFN1, MGRN1, MID1, MID2, MKRN1, MKRN2, MKRN3, MRAP, MUL1, NAIP, PARP9, PCNP, PDZRN3, PJA2, PLCD4, RBCK1, RLIM, RMND5B, RNF10, RNF103, RNF11, RNF111, RNF114, RNF115, RNF125, RNF126, RNF128, RNF13, RNF130, RNF138, RNF14, RNF144A, RNF150, RNF165, RNF166, RNF167, RNF181, RNF182, RNF25, RNF26, RNF38, RNF4, RNF41, RSPRY1, SGCE, SH3RF2, STRADB, TMBIM6, TMEM189, TOPORS, TRAF7, TRIM2, TRIM25, TRIM26, TRIM27, TRIM32, TRIM35, TRIM39, TRIM8, UBA52, UBA6, UBE2D1, UBE2D2, UBE2D3, UBE2D4, UBE2E1, UBE2E2, UBE2E3, UBE2G1, UBE2J1, UBE2K, UBE2L3, UBE2L6, UBE2N, UBE2O, UBE2Q2, UBE2R2, UBE2T, UBE2V2, UBE2W, UBE2Z, UBE4A, UBOX5, UBTD1, UEVLD, UGP2, UHRF2, WDR91, XAF1, XIAP, ZNRF1, ZNRF3
EC	Common type	RNA metabolic process, RNA splicing, Transport	ARL4A, BCAS2, CHD8, CSE1L, CTCF, CTNNBL1, KPNA1, KPNA2, KPNA4, PLAG1, PLRG1, PRPF19, RECQL, RPL22, ZNF143
EC	Common type	Synaptic transmission, Transport	ACCN2, AGAP2, C1orf116, CACNG2, CSPG4, GRIA1, GRIA2, GRIA3, GRIA4, GRIK1, GRIK3, GRIK5, GRIP1, GRIPAP1, GRM3, HOMER1, MLF1, NUMBL, PICK1, PPM1A, RYR1, SDCBP, SOX4, STOML1
EC	AD-specific type	Cellular biosynthetic process	AGL, CNTNAP4, GFPT1, GYG1, GYG2, GYS1, MAST3, NET1, PRKAB2, SH3PXD2A, STIM1, STIM2
EC	AD-specific type	Glycolysis, Metabolic process, Oxidation reduction	DGKE, DLAT, PDHA1, PDHB, PDHX, PDK3
EC	AD-specific type	Transport	ADAP1, ANP32A, CHRM3, CSNK1A1, DKC1, ERF, LYPLA2, NME1, NUP88, PPP1R14A, PTMA, SET, SETBP1, SHQ1, SYT9, THAP7, TNFRSF1B, TRPV1
EC	Early-disrupted type	Cell cycle, Protein modification process, Transport	ACTN4, ATF1, CALM3, CAMK2A, CAMK2B, CAMK2D, MYO10, TRIM3, USP6NL
EC	Early-disrupted type	Glycolysis	C16orf45, ENO2, ENSA, HK1, MAP4, SNUPN, TUBA4A, ZNF259
EC	Early-disrupted type	Axon guidance, Cellular component organization, Kinase activity	ARHGAP10, ARHGAP44, CBLL1, CCDC104, CDC42, CDC42BPA, CDC42SE1, CDC42SE2, CLIP1, CSN2, DOCK9, FGD1, FMNL2, HN1, HPS4, IQGAP1, MCF2L, MYL6B, OLFM2, OPHN1, PAK3, PAK6, PAK7, RAC1, ST13
EC	Late-disrupted type	MRNA stabilization, Protein folding, RNA catabolic process	ARCN1, ETF1, GSPT1, GSPT2, MRPL39, PABPC1, PAIP1, PAIP2, PAN2, PAN3, PCBP1, PFDN2, PRMT6, VBP1
EC	Late-disrupted type	Cell polarity, Cellular component movement, Chromatin modification, DNA repair, Growth, Kinase activity, Transcription	ACTB, ACTG1, ACTL6A, ACTR6, ACTR8, BRD8, C20orf20, CAP1, CAP2, CCDC87, CFL1, CFL2, DIP2A, DMAP1, DPCD, DSTN, E2F7, EP400, EPC1, EPC2, FKBP3, GEMIN7, H2AFV, H2AFZ, ING3, INO80, INO80B, INO80C, INO80E, KAT5, MBTD1, MCRS1, MORF4L1, MORF4L2, MRFAP1, MRFAP1L1, NCALD, NFRKB, NUFIP1, PALB2, PCBP1, PCYT1B, PIH1D1, PLA2G4A, PROM1, RUVBL1, RUVBL2, SENP6, SSH1, SSH2, TELO2, TFPT, TRIM68, TTI1, TUBA1A, VPS72, VSNL1, YEATS4, YY1, ZNF232, ZNHIT1
EC	Late-disrupted type	Cellular component organization, DNA repair, Response to stimulus	APITD1, C17orf70, ERCC1, ERCC4, FANCF, FANCG, FANCL, HES1, RMI1, RTN2, SAMD3, STRA13, UBL7
EC	Late-disrupted type	Transcription	ALDH3A2, ATP1A1, COBRA1, COX2, COX5A, COX5B, COX6B1, COX6C, FXYD1, RDBP, RPS26, SERF2, TH1L, TMEM141, TSR2, WHSC2
EC	Late-disrupted type	Biological_process, Cell adhesion, Cell differentiation, DNA replication, Growth, Kinase activity, Phosphorylation, Protein catabolic process, Protein modification process, Response to stimulus, Signaling, Small molecule metabolic process	ARL2, BEST1, C22orf39, CCNG2, CCT2, CCT3, CCT4, CCT5, CCT6A, CCT6B, CCT7, CCT8, CEP350, CLDN12, CTTNBP2, CTTNBP2NL, ECSIT, FAM40A, FAM40B, FGFR1OP, FGFR1OP2, HECTD1, IER2, IER5, IGBP1, KLHDC2, MARCH1, MCC, PDCD10, PIM1, PPME1, PPP2CA, PPP2CB, PPP2R1A, PPP2R1B, PPP2R2B, PPP2R2D, PPP2R3A, PPP2R4, PPP2R5A, PPP2R5B, PPP2R5C, PPP2R5D, PPP2R5E, PRDX2, PRR14, RAB18, RFWD2, SKA2, SKIV2L2, SLMAP, STK25, STRN, STRN3, STRN4, TCP1, TUBA8, ZCCHC8, ZNF136, ZNF295, ZRANB1
EC	Late-disrupted type	Catalytic activity, Cell adhesion, Cell death, Cellular component organization, Membrane protein ectodomain proteolysis, Notch receptor processing, Protein catabolic process, Protein processing, Proteolysis, Signaling	APBA2, APBA3, APBB2, APBB3, APP, BACE1, BGN, CIB1, CLSTN1, ICAM5, KCNIP4, NCSTN, PSEN1, PSEN2, PSENEN, SLK, YME1L1

EC	Late-disrupted type	Cell cycle, Cell division, Cellular component organization, Mitosis, Mitotic chromosome condensation	FRMD4A, NCAPD3, NCAPH2, OLFM1, SMC2, SMC4, SNAP91, TRAF3IP1
EC	Late-disrupted type	Endocytosis, Nuclear mRNA 3'-splice site recognition, Nuclear mRNA splicing, via spliceosome, RNA metabolic process, RNA splicing, Response to stimulus	ABI1, APBB1, APBB1IP, ASAP1, CHERP, COBL, CPSF6, CPSF7, CYFIP1, CYFIP2, DDX42, DDX46, DHX15, DNMBP, EVL, FMNL3, FYB, GAS7, GIN1, HTATSF1, MICALL1, NCK2, NCKAP1, PACSIN1, PACSIN2, PHF5A, PRMT2, PRPF40A, RAPH1, RBM17, RCC2, RUFY2, SF1, SF3A1, SF3A2, SF3A3, SF3B1, SF3B14, SF3B2, SF3B3, SF3B4, SF3B5, SMNDC1, TCERG1, TPM4, TSHZ1, TSHZ2, TSHZ3, VASP, WAS, WASF2, WBP11, WBP4, WIPF2, WIPF3, WWOX, YLPM1
EC	Late-disrupted type	Sensory perception of taste, Signaling	ARHGEF18, GNB1, GNB2, GNB3, GNB4, GNB5, GNG10, GNG13, GNG3, GNG4, GNG5, KCNJ3
EC	Late-disrupted type	Cell death, Cellular biosynthetic process, Histone H3 acetylation, Histone deubiquitination, Transcription	ADAT3, ANKRD12, ANLN, ATXN7, ATXN7L3, BRD2, BTAF1, CCDC101, CREM, DRAP1, ENY2, FAM48A, FOXF2, GTF2A1, GTF2A2, GTF2B, GTF2E1, GTF2E2, GTF2F1, GTF2H4, KAT2A, KLF5, KRT2, LAMB2, NFYB, NR1D2, POU3F2, SAP130, SETD7, SND1, SUPT3H, SUPT7L, TADA1, TADA3, TAF1, TAF10, TAF11, TAF12, TAF1L, TAF2, TAF3, TAF4, TAF4B, TAF5, TAF5L, TAF6, TAF7, TAF9, TAF9B, TBP, TBPL1, TCF12, THY1, TRIM24, TXNDC11, ZNF7
EC	Late-disrupted type	Anterior/posterior pattern formation, Cell proliferation, Developmental process, Transcription	BTG1, BTG2, CNOT1, CNOT10, CNOT3, CNOT7, CNOT8, HMGN1, HOPX, HOXB9, PHTF1
EC	Late-disrupted type	RNA metabolic process	NOVA1, POP1, POP4, POP5, POP7, RPP14, RPP30, RPP38, RPP40
EC	Late-disrupted type	Cell cycle, Developmental process, Interspecies interaction between organisms, Protein catabolic process	ADRM1, ANKIB1, ASPRV1, C19orf60, CKMT2, CYB5B, DERA, DLD, DSP, DUSP14, HAUS7, HERC3, HERC6, HTR1E, INSIG2, JKAMP, LENG8, MAP3K5, MB, NUB1, PAAF1, PCID2, POTEKP, PSMA1, PSMA2, PSMA4, PSMA6, PSMA7, PSMB2, PSMB3, PSMB5, PSMB6, PSMB7, PSMC1, PSMC2, PSMC3, PSMC4, PSMC5, PSMC6, PSMD1, PSMD10, PSMD11, PSMD12, PSMD13, PSMD14, PSMD2, PSMD3, PSMD4, PSMD7, PSMD8, PTPN2, RNF185, RPN1, S100A14, S100A16, SAC3D1, SHFM1, SRXN1, TXN, TXNL1, UBE3C, UBLCP1, UBQLN2, UCHL5, USP14, USP37
EC	Late-disrupted type	Apoptosis, Cell cycle, Cell proliferation, Cellular biosynthetic process, Macrophage activation, Ossification	CD74, CLEC2L, CRB1, EXT1, EXT2, FIBP, HYOU1, KIAA0528, MIF
EC	Late-disrupted type	Cellular component organization, Transport	NUP107, NUP37, NUP85, SEC13, SEC16B, SEC31A, SEH1L
EC	Late-disrupted type	Cell cycle, Cell division, Mitosis, Protein modification process	CDC42EP3, SEPT2, SEPT6, SEPT7, SEPT9, STMN1
EC	Late-disrupted type	Transport	ALDOA, ALDOC, ATP6V1E1, ATP6V1G1, ATP6V1G2, ATP6V1H
EC	Late-disrupted type	Apoptosis, Interspecies interaction between organisms, Response to stimulus, Survival gene product expression	BNIP3, BNIP3L, CD47, DOK5, HLA-DPB1, PKMYT1, ROBO2, STEAP3, TMEM11, TRIM13
EC	Late-disrupted type	Protein modification process, Transport	COG1, COG2, COG3, COG4, COG5, COG6, COG7
EC	Late-disrupted type	Attachment of GPI anchor to protein	GPAA1, PIGK, PIGS, PIGT, ULBP2
EC	Late-disrupted type	Cellular biosynthetic process, Protein modification process	DPM1, DPM2, DPM3, PIGA, PIGC, PIGH, PIGP, PIGQ
EC	Late-disrupted type	Biological_process	MPDZ, PLEKHA1, PLEKHA2
EC	Late-disrupted type	Calcium ion-dependent exocytosis, Exocytosis, Membrane fusion, Secretion, Synaptic transmission, Synaptic vesicle docking during exocytosis, Transport	BCAP29, BCAP31, CABLES2, CDK3, CDK5, CPLX1, CPLX2, DOC2A, EHD3, ITSN1, OSBP, PRRT2, SCAMP1, SCAMP2, SLC38A2, SLC6A1, SNAP23, SNAP25, SNAP29, SNTG1, SRCIN1, STX11, STX12, STX17, STX1A, STX1B, STX2, STX3, STX4, STXBP1, STXBP3, STXBP5, SV2C, SYT1, SYT3, TNFRSF21, UNC13B, VAMP1, VAMP2, VAMP3, VAMP7, VAPA, VAPB
EC	Late-disrupted type	MRNA cleavage, MRNA polyadenylation, Nuclear mRNA splicing, via spliceosome, RNA metabolic process, Transcription	CDC73, CNTNAP2, CPSF1, CPSF2, CPSF3, CPSF4, CSTF1, CSTF2, CSTF2T, CSTF3, CTR9, FIP1L1, HSF2BP, LEO1, NOL8, PAF1, RRAGA, RRAGB, RRAGC, RRAGD, RTF1, SUPT6H, SYMPK, TCEA1, TTC37, WDR61
EC	Late-disrupted type	Angiogenesis, Cell differentiation, Cell migration, Cell morphogenesis, Cell proliferation, Cell shape, Cellular biosynthetic process, Cellular component organization, DNA replication, Developmental process, Fatty acid beta-oxidation, Feeding behavior, Germ cell programmed cell death, Glucose homeostasis, Interspecies interaction between organisms, Kinase activity, Mast cell degranulation, Metabolic process, Neutrophil chemotaxis, Organ morphogenesis, Phosphorylation, Protein modification process, Response to stimulus, Secretion, Signaling, Small molecule metabolic process, Transport, Vasculogenesis	ABI2, ABL1, ABL2, AEBP1, AP3S1, AREG, ARHGAP17, ARHGAP32, BBS10, BCR, BDNF, BLNK, C21orf91, CAV1, CAV2, CBLB, CD164, CD22, CD226, CD24, CD2AP, CEND1, CENPV, CORO2B, CRKL, CSF1R, CSK, CSRP1, CUTA, DNAJC4, DOK2, EFNB2, EGF, EGFR, EPHB1, EPHB2, EPOR, ERBB3, ERBB4, ERRFI1, FAM59A, FCER1G, FCGR2A, FIGF, FLOT2, FYN, GAB1, GHR, GP6, GPM6B, GRB10, GRB14, GRB2, HCLS1, HCST, IL4R, INSR, IRS1, IRS2, ITIH4, KDR, KHDRBS1, KIAA0319, KIT, KITLG, LILRB4, LYN, MAP4K3, MAP4K5, MAST1, MCAM, MET, MICAL1, MINK1, NCK1, NCK2, NEDD9, NRG1, NRG2, NTRK2, OPRK1, PAG1, PDCD6IP, PDGFRB, PIK3AP1, PIK3C2B, PIK3R1, PILRA, PLCG2, PLD2, POMP, PRAM1, PRCC, PTK2, PTPN11, PTPN23, PTPN6, RASA1, RET, RPS6KB2, SDC3, SEMA6A, SH2B1, SH2B2, SH3BGRL, SH3KBP1, SH3RF1, SHC1, SHE, SKAP2, SLA, SLAMF6, SLC1A2, SLC9A3R1, SNX4, SOCS2, SOCS3, SOCS7, SORBS1, SORBS2, SOS1, SOS2, SPARCL1, STK32C, SYK, SYP, TCEAL8, TGFB1I1, TLN1, TYK2, UBA52, VAV2, VCL, YES1, YTHDC1, ZDHHC16
EC	Late-disrupted type	Gene silencing by RNA, Translation	ADI1, AGK, ARF4, ATP5I, DBT, EIF2C1, EIF2C3, EIF2C4, EIF4B, IPO8, PTS, RPL27, RPL27A, RPL35, SLC25A1, SLC25A22, SPIN1, SUCLA2, TNRC6A, TNRC6B

EC	Late-disrupted type	Cell proliferation, Developmental process, Kinase activity, Lipid metabolic process, Receptor-mediated endocytosis, Secretion, Transport	ADRB1, ANKS1B, APOE, CNPY2, DAB1, GIPC1, LDLRAP1, LPL, LRP1, LRP2, LRP2BP, LRP8, LRPAP1, MYLIP, NOS1AP, PLTP, RELN, SCN3A, SNX17, SORL1, SORT1, SYN2, SYN3, SYNJ2BP, TYRP1, VLDLR
EC	Late-disrupted type	Cell cycle, Cell division, Cellular component organization, Meiosis, Mitosis, Mitotic sister chromatid cohesion	IQCB1, MXI1, PDS5A, PDS5B, RPGR, SMC1A, SMC3, STAG2, STAG3, WAPAL
EC	Late-disrupted type	Cellular biosynthetic process, Nuclear mRNA splicing, via spliceosome, RNA metabolic process, Signaling, Transcription	ARMCX3, C15orf44, C7orf26, CCNC, CDK19, CDK8, CPSF3L, CTDP1, DDX26B, FKBP14, GPN1, GPN3, GTF2F2, INTS1, INTS12, INTS2, INTS4, INTS5, INTS6, INTS7, INTS8, KIF1A, LRCH2, MED1, MED10, MED11, MED12, MED13L, MED14, MED15, MED16, MED19, MED20, MED21, MED22, MED24, MED27, MED28, MED29, MED4, MED6, MED7, MED8, MED9, MEIG1, OBFC2A, OBFC2B, PIH1D1, POLR2B, POLR2C, POLR2D, POLR2E, POLR2F, POLR2G, POLR2H, POLR2I, POLR2J, POLR2K, POLR2L, POLR3A, POLR3B, PPARGC1A, QKI, RPAP2, SHD, SREBF1, STK19, SYT5, TADA2B, TRIP4, ZC3H13, ZMYND8, ZNF592
EC	Late-disrupted type	RNA catabolic process, RNA metabolic process, Transport	C21orf56, DCP1A, DCP1B, DCP2, DOM3Z, EDC4, EXOSC10, PARN, PTGES2, RPE, RPS15, SMG1, TARDBP, UPF1, UPF2, UPF3B, USP16, XRN1, XRN2
HIP	Common type	Biological_process, Transcription	ALDH3A2, ATP1A1, C19orf29, COBRA1, COX2, COX5A, COX5B, COX6B1, COX6C, RDBP, RPS26, SERF2, TH1L, TMEM141, TSR2, WHSC2
HIP	Common type	Developmental process, Spermatogenesis, Transcription	ARNT2, BACH2, BCL6, DDX6, DNAH9, DROSHA, EBF1, EBF3, EPHB6, KDM2A, KIF13B, LPAR2, LPAR4, OLA1, OSBPL1A, PARP12, PCDH9, PIKFYVE, PPAP2B, SENP7, SMO, SPTLC2, WDR35, WNT16, ZNF423, ZNF443
HIP	Common type	Cellular component organization, Cerebral cortex cell migration, Neuron migration, Peroxisome fission, Peroxisome membrane biogenesis, Transport	DDO, FIS1, GDAP1, PEX10, PEX11B, PEX12, PEX13, PEX14, PEX16, PEX19, PEX2, PEX3, PEX5, PEX7, SLC25A17
HIP	Common type	DNA replication, Biological_process, Cell adhesion, Cell differentiation, Kinase activity, Phosphorylation, Protein catabolic process, Protein modification process, Response to stimulus, Signaling, Small molecule metabolic process	ABCB1, APOD, ARL2, BEST1, C22orf39, CCNG2, CCT2, CCT3, CCT4, CCT5, CCT6A, CCT6B, CCT7, CCT8, CEP350, CLDN12, CTTNBP2, CTTNBP2NL, FABP5, FAM122A, FAM40A, FAM40B, FGFR1OP, FGFR1OP2, HECTD1, IER2, IER5, IGBP1, KLHDC2, MARCH1, MCC, MTHFD1, PDCD10, PIM1, PPFIA1, PPFIA2, PPME1, PPP2CA, PPP2CB, PPP2R1A, PPP2R1B, PPP2R2B, PPP2R2D, PPP2R3A, PPP2R4, PPP2R5A, PPP2R5B, PPP2R5C, PPP2R5D, PPP2R5E, PPP4C, PPP4R1, PPP4R2, PPP4R4, PRDX2, PRR14, RAB18, RBM7, RFWD2, SERTAD4, SKA2, SKA3, SKIV2L2, SLMAP, SMEK1, STK24, STK25, STRN, STRN3, STRN4, TCP1, TIPRL, TUBA8, WDR81, ZBTB24, ZCCHC8, ZNF136, ZNF295, ZRANB1
HIP	Common type	Cell cycle, Cell differentiation, Developmental process, Peptide cross-linking, Protein catabolic process, Protein modification process	ADRM1, ALB, ANKIB1, ASPRV1, C19orf60, CASP14, CKMT2, CSTA, CYB5B, DERA, DLD, DSP, DUSP14, HAUS7, HERC3, HERC4, HERC6, HTR1E, INSIG2, JKAMP, LENG8, LPCAT1, NUB1, PAAF1, PCID2, PCK1, POTEKP, PSMA1, PSMA2, PSMA4, PSMA5, PSMA6, PSMA7, PSMB1, PSMB2, PSMB3, PSMB5, PSMB6, PSMB7, PSMC1, PSMC2, PSMC3, PSMC4, PSMC5, PSMC6, PSMD1, PSMD10, PSMD11, PSMD12, PSMD13, PSMD14, PSMD2, PSMD3, PSMD4, PSMD8, PTPN2, RAD23B, RNF185, RPN1, S100A14, S100A16, SAC3D1, SERPINB12, SHFM1, SLC25A22, SRXN1, TGM3, TIMP2, TOMM70A, TXNL1, UBE3C, UBLCP1, UBQLN2, UCHL5, USP14, USP37, USP53
HIP	Common type	RNA metabolic process	NOVA1, POP1, POP4, POP5, POP7, RPP14, RPP25, RPP30, RPP38, RPP40
HIP	Common type	Cell death, Cellular biosynthetic process, Histone H3 acetylation, Histone deubiquitination, Transcription	ADAM33, ADAT3, ANKRD12, ANLN, ATF7IP, ATXN7, ATXN7L3, BRD2, BTAF1, CCDC101, CREG1, CREM, DRAP1, ENY2, FOXF2, GTF2A1, GTF2A2, GTF2B, GTF2E1, GTF2E2, GTF2F1, GTF2H4, IGF2R, KAT2A, KLF5, NFYB, NR1D2, PAX3, PAX6, POU3F2, SAP130, SETD7, SUPT3H, SUPT7L, TADA1, TADA3, TAF1, TAF10, TAF11, TAF2, TAF3, TAF4, TAF4B, TAF5, TAF5L, TAF6, TAF7, TAF8, TAF9, TAF9B, TBP, TBPL1, THY1, TXNDC11, WDHD1
HIP	Common type	Cellular component organization, Transport	NUP107, NUP160, NUP37, NUP85, SEC13, SEC31A, SEH1L
HIP	Common type	Biological_process	AASS, ABCB7, ACADVL, ACOT9, AGK, ALDH18A1, ALDH1L2, ATP5H, C14orf169, C6orf203, C7orf30, CECR5, CHCHD1, CPT2, DDX28, DDX56, DHX37, ERAL1, FASTKD2, FECH, FTSJ3, FXN, GLUD1, GRSF1, HARS2, ICT1, ISCA1, MCAT, MMAB, MRPL1, MRPL10, MRPL11, MRPL13, MRPL14, MRPL15, MRPL16, MRPL17, MRPL18, MRPL2, MRPL22, MRPL23, MRPL24, MRPL27, MRPL28, MRPL3, MRPL32, MRPL37, MRPL40, MRPL41, MRPL43, MRPL45, MRPL46, MRPL47, MRPL48, MRPL49, MRPL51, MRPL54, MRPL9, MRPS10, MRPS11, MRPS14, MRPS15, MRPS16, MRPS18A, MRPS2, MRPS21, MRPS23, MRPS24, MRPS25, MRPS28, MRPS30, MRPS34, MRPS35, MRPS5, MRPS6, MRPS7, MRPS9, MRRF, MTERF, MTERFD1, MTERFD2, MTG1, MTIF2, MUT, NDUFA9, NDUFS3, NDUFS8, NGRN, OXA1L, PARS2, PCCB, PMPCA, PMPCB, PNPT1, PRDX5, PTCD1, PUS1, PUSL1, RPL37, RPUSD3, SLC25A18, SUCLA2, SUPV3L1, TARS2, TEX10, TRUB2, USP42, VDAC3, WBSCR16, YARS2, YME1L1, YTHDC2
HIP	Common type	Cellular biosynthetic process, Protein modification process	DPM1, DPM2, DPM3, PIGA, PIGC, PIGH, PIGP, PIGQ

HIP	Common type	RNA metabolic process	IMP3, IMP4, LIMS1, LPXN, MPHOSPH10
HIP	Common type	Attachment of GPI anchor to protein	GPAA1, PIGK, PIGS, PIGT, ULBP2
HIP	Common type	Biological_process	MPDZ, PLEKHA1, PLEKHA2
HIP	Common type	RNA metabolic process, MRNA cleavage, MRNA polyadenylation, Nuclear mRNA splicing, via spliceosome, Transcription	CDC73, CNTNAP2, CPSF1, CPSF2, CPSF3, CPSF4, CSTF1, CSTF2, CSTF2T, CSTF3, CTR9, DDX46, FIP1L1, HSF2BP, LEO1, MLL, MTM1, NIP7, NOL8, PAF1, RRAGA, RRAGB, RRAGC, RRAGD, RTF1, SUPT6H, TCEA1, TTC37, WDR61
HIP	Common type	Cell proliferation, Developmental process, Kinase activity, Lipid metabolic process, Receptor-mediated endocytosis, Secretion, Transport	ADRB1, AHRR, ANKRA2, ANKS1B, APBA2, APOE, CLU, CNPY2, DAB1, GIPC1, KIF1B, LDLRAP1, LPL, LRP1, LRP2, LRP2BP, LRP8, LRPAP1, MMP25, MYLIP, MYO6, NECAB3, NOS1AP, PLTP, RASD1, RELN, SCN3A, SDC4, SNX17, SORL1, SORT1, SYN2, SYN3, SYNJ2BP, TYRP1, VLDLR
HIP	Common type	RNA metabolic process, Cellular biosynthetic process, Nuclear mRNA splicing, via spliceosome, Signaling, Transcription	ARMCX3, C15orf44, C7orf26, CCHCR1, CCNC, CDK19, CDK8, CPSF3L, CTDP1, DDX26B, EGR2, FKBP14, GPN1, GPN3, GTF2F2, INTS1, INTS12, INTS2, INTS4, INTS5, INTS6, INTS7, INTS8, LRCH2, MED1, MED10, MED11, MED12, MED13, MED13L, MED14, MED15, MED16, MED17, MED19, MED20, MED21, MED22, MED24, MED27, MED28, MED29, MED31, MED4, MED6, MED7, MED8, MED9, MEIG1, MYEF2, NAIF1, OBFC2A, OBFC2B, ORAI2, PDRG1, POLR2B, POLR2C, POLR2D, POLR2E, POLR2F, POLR2G, POLR2H, POLR2I, POLR2J, POLR2K, POLR2L, POLR3A, POLR3B, PPARGC1A, QKI, RPAP1, RPAP2, RPAP3, SHD, SREBF1, SYT5, TADA2B, TRIM11, TRIP4, UXT, ZC3H13, ZNF592
HIP	Common type	DNA repair, Apoptosis, Protein catabolic process, Protein metabolic process, Protein modification process, Proteolysis, Signaling	AKR1C3, ARIH1, ARIH2, BCL10, BFAR, BIRC2, BIRC3, BIRC6, BMI1, C6orf165, C9orf89, CADPS2, CASP7, CBX4, CBX7, CD40LG, CGRRF1, CNOT4, DDX58, DIABLO, DNAJC2, DTX1, DTX3L, DZIP3, EIF4E2, FAM178B, FLT3, GPR108, HLTF, HTRA2, ISG15, KIAA1107, KIAA1797, L3MBTL2, LNX2, LOC147791, LRSAM1, LTN1, MAGEL2, MAP3K1, MARCH5, MARCH7, MC1R, MFN1, MGRN1, MIB2, MID1, MID2, MKRN1, MKRN2, MKRN3, MUL1, PCGF2, PCGF6, PCNP, PDZRN3, PEG10, PEG3, PHC1, PHC3, PJA2, RBCK1, RC3H2, RFWD3, RING1, RLIM, RMND5B, RNF10, RNF103, RNF11, RNF111, RNF113A, RNF114, RNF115, RNF125, RNF126, RNF128, RNF13, RNF130, RNF138, RNF14, RNF144A, RNF150, RNF165, RNF166, RNF167, RNF170, RNF181, RNF182, RNF2, RNF25, RNF26, RNF4, RNF41, RNFT1, RSPRY1, SFMBT1, SGCE, SIAH1, STAM2, TMBIM6, TMEM189, TOPORS, TRAF7, TRIM2, TRIM25, TRIM26, TRIM27, TRIM32, TRIM35, TRIM39, TRIM46, TRIM8, UBA6, UBE2D2, UBE2D3, UBE2D4, UBE2E1, UBE2E2, UBE2E3, UBE2G1, UBE2J2, UBE2K, UBE2L3, UBE2L6, UBE2N, UBE2O, UBE2Q2, UBE2R2, UBE2T, UBE2U, UBE2V2, UBE2W, UBE2Z, UBE4A, UEVLD, UGP2, UHRF2, VPS41, WDR91, XAF1, XIAP, ZNRF1, ZNRF2, ZNRF3
HIP	AD-specific type	Cell adhesion, Cellular component organization	EFNA5, EPHA7, EPHB6, MLLT4, MYO16, NLGN1, NRXN1, NRXN2, NRXN3, PVRL1, PVRL3, RAB5B, RIN1, RRAS2, SIPA1L1, SSX2IP, SYT13
HIP	AD-specific type	Cell differentiation, Developmental process	ANK2, CENPF, CEP170, DIXDC1, FAM190A, KALRN, MRC2, NDEL1, ZNF10, ZNF17, ZNF197, ZNF211, ZNF91
HIP	AD-specific type	Cellular biosynthetic process	AGL, CNTNAP4, GFPT1, GYG1, GYG2, GYS1, MAST3, NET1, PRKAB2, SH3PXD2A, STIM1, STIM2, TRIM7
HIP	AD-specific type	Cellular component organization, Chromatin modification, Chromatin remodeling, Response to stimulus, Signaling, Transcription	ADNP, ARID1A, ARID1B, ARID2, BATF3, BCL7A, BCL7C, BRD7, CARM1, CHD7, DACH2, DAZAP1, DPF2, HSP90B1, IFNAR2, IRF1, IRF9, ITCH, LAPTM5, MYEF2, PABPN1, PBRM1, PGR, PHF10, REST, SALL3, SMARCA4, SMARCC1, SMARCC2, SMARCD1, SMARCD2, SMARCE1, SNX18, STAT2, ZBTB20, ZMIZ2
HIP	AD-specific type	Apoptosis, Cell cycle, Cell proliferation, Chemotaxis, Exocytosis, Organ morphogenesis, Protein modification process, Signaling	ABCC8, ATPIF1, BRAF, CNKSR1, CNKSR2, DGKZ, EXOC2, EXOC8, FTL, HRAS, KRAS, KSR2, MAP1S, MAP2K1, MRAS, MRPL36, NRAS, PEBP1, PEBP4, PIK3CA, PLCE1, PRKRIR, RAF1, RALA, RAP1A, RAP1GDS1, RAP2A, RAP2B, RAPGEF2, RAPGEF4, RAPGEF5, RASD2, RASSF2, RASSF4, RASSF5, RGL1, RGL2, RHEB, RPS20, RRAS, RUNDC3A, SAV1, SERGEF, SHOC2, STK3, STK4, TM7SF2, VARS2, ZHX2, ZHX3
HIP	AD-specific type	Axon guidance, Cellular component organization, Chemotaxis, Kinase activity	ARHGAP10, ARHGAP26, ARHGAP44, ARHGEF25, ARHGEF7, ARL2BP, BAIAP2, BNIP2, BNIPL, CBLL1, CCDC104, CCDC59, CDC42, CDC42BPA, CDC42SE1, CDC42SE2, CLIP1, CSN2, DNMBP, DOCK2, DOCK9, FGD1, FMNL2, HACE1, HN1, HPS4, IQGAP1, KIAA2026, LATS1, MAP3K4, MCF2L, MYL6B, MYO9A, NCKAP1, OLFM2, OPHN1, PAK2, PAK3, PAK6, PAK7, RAC1, ST13, WASF1, WIPF3, ZNF234
HIP	Early-disrupted type	Adult locomotory behavior, Apoptosis, Cell differentiation, Cellular component organization, Protein folding, Protein modification process, Response to stimulus, Signaling	AHSA2, AIP, APBB2, APP, ASNA1, BAG1, BAG2, BAG3, BAG4, BAG5, BGN, DNAJB1, DRD5, DYNLT1, EIF2AK1, FABP3, FAM189A2, FKBP5, GNA12, GNA13, GPR37, GRK5, GUCY1B3, HSP90AA1, HSPA14, HSPA1A, HSPA4, HSPA8, HSPBP1, LRRK2, MAP3K3, MAP3K5, MAPT, MCF2, MST1R, NDUFB6, NQO1, OXTR, PPIL2, PPP5C, PRNP, RGNEF, SEC61B, SERP1, SNCA, SNCB, SOD1, SRP54, STIP1, STMN1, STUB1, TNNI3K, TRIAP1
HIP	Early-disrupted type	Oxidation reduction	CCDC86, DLD, DLST, NAP1L1, OGDH, RUSC2
HIP	Early-disrupted type	Apoptosis, Interspecies interaction between organisms, Response to stimulus, Survival gene product expression	BNIP3, BNIP3L, DOK5, HLA-DPB1, ROBO2, STEAP3, TMEM11, TRIM13
HIP	Late-disrupted type	MRNA stabilization, Protein folding, RNA catabolic process	ARCN1, ETF1, GSPT1, GSPT2, MRPL39, PABPC1, PAIP1, PAIP2, PAN2, PAN3, PCBP1, PFDN2, PRMT6, VBP1
HIP	Late-disrupted type	Transport	FAM125B, HCRP1, PPFIBP2, TSG101, VPS28, VPS37A, VPS37B, VPS37C

HIP	Late-disrupted type	Protein catabolic process, Protein modification process, Response to stimulus	ASB8, ATRN, BRWD1, C12orf10, CAND1, CDC34, COMMD1, COPS2, COPS3, COPS4, COPS5, COPS6, COPS7A, CORO7, CRBN, CUL2, CUL3, CUL4B, CUL7, DCAF10, DCAF12, DCAF4, DCAF5, DCAF8, DCUN1D1, DDB1, DDB2, DET1, DIO2, EDN1, ENC1, ERCC8, FAU, FBXL3, FBXL4, FBXO11, FBXO18, FBXO22, FBXO3, FBXO31, FBXO34, FBXO4, FBXO42, FBXO7, FBXW9, GPS1, IRF8, KATNA1, KLHDC10, KLHDC5, KLHL13, KLHL15, KLHL18, KLHL21, KLHL22, KLHL24, KLHL8, LRRC14, MAPRE1, MYEOV2, NUDCD3, PFKFB3, POC1B, PTGS2, RBX1, RCBTB2, ROGDI, SHISA5, SKP1, SMU1, SOCS6, SPSB3, TCEB1, TCEB2, TCEB3, TUBD1, UBR5, UBXN7, WDTC1, ZMYM4, ZMYM6, ZYG11B
HIP	Late-disrupted type	Sensory perception of taste, Signaling	ARHGEF18, GNB1, GNB2, GNB3, GNB4, GNB5, GNG10, GNG11, GNG13, GNG3, GNG4, GNG5, KCNJ3, RGS6
HIP	Late-disrupted type	Protein modification process, Transport	COG1, COG2, COG3, COG4, COG5, COG6, COG7
HIP	Late-disrupted type	Cell cycle, Cell proliferation, Cellular biosynthetic process, DNA repair, Kinase activity, Response to stimulus, Sensory perception of sound, Transcription	CCNH, CDK7, ERCC3, ERCC5, GTF2H1, GTF2H2, GTF2H3, GTF2H5, MNAT1
HIP	Late-disrupted type	Cell cycle, DNA repair, Double-strand break repair, Metabolic process, Protein catabolic process, Protein modification process, Response to stimulus	ATP5O, BAG5, BRCC3, BRE, CPNE1, DCUN1D1, DCUN1D4, DHRS7B, F7, FAM175B, NAE1, PYCRL, SHMT2, SSR3, THNSL1, TMX3, UBA3, UBE2M, UGGT2, UIMC1
HIP	Late-disrupted type	Endocytosis, Signaling	AGFG1, AGFG2, AP2A2, DPYSL2, EPN1, EPS15, EPS15L1, FCHO2, GJB1, GTF3A, NAGPA, NUMB, OCLN, RALBP1, REPS2, SNAP91
SFG	Common type	Transcription	ALDH3A2, ATP1A1, COBRA1, COX2, COX4I1, COX5A, COX5B, COX6B1, COX6C, DNPEP, RDBP, SERF2, TH1L, TMEM141, WHSC2
SFG	Common type	Cell cycle, Developmental process, Interspecies interaction between organisms, Protein catabolic process, Proteolysis	ADRM1, ALDOA, ANKIB1, ASAH1, ASPRV1, C19orf60, CKMT2, CPT1A, CYB5B, DERA, DLD, DONSON, DUSP14, HAUS7, HTR1E, INSIG2, IVL, JKAMP, LDHA, LENG8, LPCAT1, MAP3K5, MB, NUB1, PAAF1, PCID2, PCK1, PSMA1, PSMA2, PSMA3, PSMA4, PSMA5, PSMA6, PSMA7, PSMB1, PSMB2, PSMB3, PSMB5, PSMB6, PSMB7, PSMC1, PSMC2, PSMC3, PSMC4, PSMC5, PSMC6, PSMD1, PSMD10, PSMD11, PSMD12, PSMD13, PSMD14, PSMD2, PSMD3, PSMD4, PSMD7, PSMD8, PTPN2, RAD23B, RNF185, RPN1, S100A14, S100A16, SAC3D1, SHFM1, SLC25A22, SRPRB, SRXN1, TIMP2, TOMM70A, TPI1, TXN, TXNL1, UBE3C, UBLCP1, UBQLN2, UCHL5, USP14, USP37
SFG	Common type	Transport	KIAA1549, MBD5, MYO5B, RAB11A, RAB11B, RAB11FIP1, RAB11FIP2, RAB11FIP3, RAB11FIP4, RAB11FIP5, REPS1
SFG	Common type	Cellular component organization, Cerebral cortex cell migration, Neuron migration, Peroxisome fission, Peroxisome membrane biogenesis, Transport	DDO, FIS1, GDAP1, PEX10, PEX11B, PEX12, PEX13, PEX14, PEX16, PEX19, PEX2, PEX3, PEX5, PEX7, SLC25A17
SFG	Common type	DNA replication, Biological_process, Cell adhesion, Cell differentiation, Growth, Kinase activity, Phosphorylation, Protein catabolic process, Protein modification process, Response to stimulus, Signaling, Small molecule metabolic process	ANKLE2, ARL2, BEST1, C22orf39, CCNG2, CCT2, CCT3, CCT4, CCT5, CCT6A, CCT6B, CCT7, CCT8, CEP350, CLDN12, CTTNBP2, CTTNBP2NL, FAM40A, FAM40B, FGFR1OP, FGFR1OP2, HECTD1, IER2, IER5, IGBP1, KLHDC2, MARCH1, MCC, MST4, PDCD10, PIM1, PPFIA1, PPFIA2, PPME1, PPP2CA, PPP2CB, PPP2R1A, PPP2R1B, PPP2R2B, PPP2R2D, PPP2R3A, PPP2R4, PPP2R5A, PPP2R5B, PPP2R5C, PPP2R5D, PPP2R5E, PRDX2, PRR14, RAB18, RFWD2, SERTAD4, SIKE1, SIPA1, SKA2, SKIV2L2, SLMAP, STK25, STRN, STRN4, TCP1, TUBA8, ZCCHC8, ZNF136, ZNF295, ZRANB1
SFG	Common type	Cellular biosynthetic process, Histone H3 acetylation, Histone deubiquitination, Transcription	ADAM33, ANKRD12, ANLN, ASF1A, ATF7, ATF7IP, ATXN7, ATXN7L3, BTAF1, CCDC101, CREB3L4, CREM, DRAP1, ENY2, FAM48A, FOXF2, GTF2A1, GTF2A2, GTF2B, GTF2E1, GTF2E2, GTF2F1, GTF2H4, GTF3C2, GTF3C3, GTF3C5, GTF3C6, KAT2A, KLF5, MKX, NFYB, NR1D2, POLR3C, POLR3F, POU3F2, SAP130, SETD7, SNAPC2, SND1, SUPT3H, SUPT7L, TADA1, TADA3, TAF1, TAF10, TAF11, TAF12, TAF1L, TAF2, TAF3, TAF4, TAF4B, TAF5, TAF5L, TAF6, TAF7, TAF8, TAF9, TAF9B, TBP, TBPL1, TCF12, THY1
SFG	Common type	Chromatin silencing, Protein modification process, Transcription	AK5, CMYA5, MTMR2, NUCKS1, PGAP1, SBF1, SIRT3, SIRT5, TRIM46, UGDH, UQCRH
SFG	Common type	Developmental process, Response to stimulus, Translation	ADRA2C, EIF2B1, EIF2B2, EIF2B3, EIF2B4, EIF2B5, EIF2S1, MRPL4, NDUFB8, PDIA4, PIM2
SFG	Common type	Metabolic process	BEND5, CTNNBIP1, CTSA, GLB1, NACAP1, NEU1

SFG	Common type	Biological_process	AASS, ABCB7, ACADVL, ACOT9, AGK, ALAS2, ALDH18A1, ALDH1L2, ATP5H, C14orf169, C6orf203, C7orf30, CECR5, CHCHD1, CPNE3, CPT2, DDX28, DDX56, ERAL1, FASTKD2, FECH, FTSJ3, FXN, GLUD1, GRSF1, GTPBP10, HARS2, ICT1, ISCA1, MCAT, MMAB, MRPL1, MRPL10, MRPL11, MRPL13, MRPL14, MRPL15, MRPL16, MRPL17, MRPL18, MRPL19, MRPL2, MRPL22, MRPL23, MRPL24, MRPL27, MRPL28, MRPL3, MRPL32, MRPL37, MRPL40, MRPL41, MRPL43, MRPL45, MRPL46, MRPL47, MRPL48, MRPL49, MRPL50, MRPL51, MRPL9, MRPS10, MRPS11, MRPS14, MRPS15, MRPS16, MRPS18A, MRPS2, MRPS21, MRPS23, MRPS24, MRPS25, MRPS28, MRPS30, MRPS34, MRPS35, MRPS5, MRPS6, MRPS7, MRPS9, MRRF, MTERF, MTERFD1, MTERFD2, MTG1, MUT, NDUFA9, NDUFS1, NDUFS3, NGRN, OXA1L, PARS2, PCCB, PMPCA, PMPCB, PNPT1, PRDX5, PTCD1, PUS1, PUSL1, RPL37, RPUSD3, SLC25A18, SSBP1, SUCLA2, SUPV3L1, TARS2, TEX10, TRUB2, USP42, VDAC3, YME1L1, YTHDC2
SFG	Common type	Attachment of GPI anchor to protein	GPAA1, PIGK, PIGS, PIGT, ULBP2
SFG	Common type	Transport	ALDOA, ALDOC, AP2B1, ATP6V1E1, ATP6V1G1, ATP6V1G2, ATP6V1H, LDLRAP1, MEA1
SFG	Common type	Cellular component organization, Kinase activity, Localization, Response to stimulus, Transcription	ACD, C1orf63, CCDC147, CLK3, DDX3Y, FOXJ3, GRPEL1, KIAA2022, MACROD2, MCCC2, NANOG, PAK1IP1, PCMT1, PLOD1, PLOD2, POT1, RASGEF1C, RPS7, RSBN1L, SALL1, SEC16A, TDRD6, TERF1, TERF2IP, TINF2, TNKS2, YTHDF1, YTHDF3, ZC3HAV1L, ZNF281
SFG	Common type	Cell proliferation, Cellular component organization, Kinase activity, Lipid metabolic process, Receptor-mediated endocytosis, Secretion, Transport	ANKS1B, APBA2, APBB2, APBB3, APLP2, APOE, APP, BGN, CLSTN1, CLU, CNPY2, DAB1, F9, GIPC1, GULP1, LDLRAP1, LPL, LRP1, LRP2, LRP2BP, LRP8, LRPAP1, MYLIP, NOS1AP, PDXDC1, PLTP, RELN, SCN3A, SDC4, SLK, SNX17, SORL1, SORT1, SYN2, SYN3, SYNJ2BP, TPBG, TYRP1, VLDLR
SFG	Common type	DNA repair, Protein catabolic process, Protein metabolic process, Protein modification process, Response to stimulus	ARIH1, ARIH2, BFAR, CADPS2, CHFR, CNOT4, DDX58, DTX1, DTX3L, DZIP3, EIF4E2, EML4, EPS15, FAM178B, FCHO2, FUT4, GPR108, HLTF, INF2, ISG15, KIAA1107, KIAA1797, LNX2, LOC147791, LONRF1, LRSAM1, LTN1, MAGEL2, MAP3K1, MARCH5, MARCH7, MC1R, MFN1, MGRN1, MIB1, MID1, MID2, MKRN1, MKRN2, MKRN3, MRAP, MUL1, NAGPA, PARP9, PCNP, PDZRN3, PJA2, PLCD4, RBCK1, RLIM, RMND5B, RNF10, RNF103, RNF11, RNF111, RNF114, RNF115, RNF125, RNF126, RNF128, RNF13, RNF130, RNF138, RNF14, RNF144A, RNF150, RNF152, RNF165, RNF166, RNF167, RNF181, RNF182, RNF216, RNF25, RNF26, RNF38, RNF4, RNF43, RNF8, RSPRY1, SGCE, SH3RF2, STAM2, TMBIM6, TMEM189, TOPORS, TRAF7, TRIM11, TRIM2, TRIM25, TRIM26, TRIM27, TRIM32, TRIM35, TRIM39, UBA52, UBA6, UBE2D1, UBE2D2, UBE2D3, UBE2D4, UBE2E1, UBE2E2, UBE2E3, UBE2G1, UBE2J1, UBE2J2, UBE2K, UBE2L3, UBE2L6, UBE2N, UBE2O, UBE2R2, UBE2T, UBE2V2, UBE2W, UBE2Z, UBE4A, UBOX5, UBTD1, UEVLD, UGP2, UHRF2, WDR91, ZNRF1, ZNRF3
SFG	Common type	RNA metabolic process, Cellular biosynthetic process, Nuclear mRNA splicing, via spliceosome, Signaling, Transcription	ARMCX3, C15orf44, C7orf26, CCHCR1, CCNC, CDC40, CDK19, CDK8, CPSF3L, CTDP1, DDX26B, DOCK6, EGR2, FKBP14, GPN1, GPN3, GTF2F2, INTS1, INTS12, INTS2, INTS3, INTS4, INTS5, INTS6, INTS7, INTS8, LRCH2, LUZP1, MED1, MED10, MED11, MED12, MED13, MED13L, MED14, MED15, MED16, MED17, MED19, MED20, MED21, MED22, MED24, MED27, MED28, MED29, MED31, MED4, MED6, MED7, MED8, MED9, MEIG1, MYEF2, OBFC2A, OBFC2B, PDRG1, PIH1D1, POLR2B, POLR2C, POLR2D, POLR2E, POLR2F, POLR2G, POLR2H, POLR2I, POLR2J, POLR2K, POLR2L, POLR3A, POLR3B, POLR3D, POLR3H, POLR3K, PPARGC1A, QKI, RGPD1, RPAP1, RPAP2, RPAP3, SHD, SLFN11, SREBF1, STK19, SYT5, TADA2A, TADA2B, TELO2, TRIM11, TRIP4, TTI1, TWIST2, UACA, ZC3H13, ZMYND8, ZNF592
SFG	Early-disrupted type	Axon guidance, Cellular component organization, Kinase activity	ARHGAP10, ARHGAP26, ARHGAP44, ARHGEF25, ARHGEF7, BAIAP2, BNIP2, CBLL1, CCDC104, CDC42, CDC42BPA, CDC42SE1, CDC42SE2, CLIP1, DOCK9, FAM161A, FGD1, FMNL2, HN1, HPS4, IQGAP1, MCF2L, MYL6B, MYO9A, OLFM2, OPHN1, PAK3, PAK6, PAK7, PKN3, PPM1F, RAC1, ST13, VRK2, ZNF234
SFG	Early-disrupted type	RNA catabolic process, RNA metabolic process, Transport	DCP1A, DCP1B, DCP2, DOM3Z, EDC4, EIF5A, EXOSC10, MAP1A, PARN, PTGES2, RPE, RPS15, SMG1, TARDBP, UPF1, UPF2, UPF3B, XRN1, XRN2
SFG	Late-disrupted type	Actin filament polymerization, Cellular component movement	ACTR3, ACTR3B, ARAP1, ARPC1A, ARPC1B, ARPC2, ARPC3, ARPC5, ARPC5L, CRB2, CTTN, DNM3, FER, KCTD12, MLC1, MYLK, PSD3, TMF1, WASF3
SFG	Late-disrupted type	Oxidation reduction	CCDC86, DLD, DLST, NAP1L1, OGDH, RUSC2
SFG	Late-disrupted type	Protein targeting to mitochondrion, Transport	DNAJC19, EFHD1, GPM6A, PAM16, PGRMC1, SFXN1, TIMM17A, TIMM44
SFG	Late-disrupted type	Cellular biosynthetic process, Fatty acid oxidation, Glycolysis, Kinase activity, Signaling	ALDH7A1, APEH, ATG13, BRSK1, BRSK2, C12orf44, CBR1, EIF5B, ETFA, FARSB, FKBP4, FNIP1, GANAB, GFPT1, HSD17B4, LONP1, NAA15, PA2G4, PGK1, PIK3C2A, PRKAA1, PRKAB2, PRKAG1, PRKAG2, RB1CC1, SFXN1, SH3GLB2, SH3PXD2A, SIK2, SIK3, SND1, TARS, UBA5, ULK1
SFG	Late-disrupted type	Transport	ARL6, ARL6IP1, ARL6IP4, ARL6IP5, ARL6IP6, HMGCL, SLC1A1
SFG	Late-disrupted type	Transcription, Transport	MLX, MLXIP, MLXIPL
SFG	Late-disrupted type	Cell cycle, Cell division, Cell proliferation, Mitosis, Mitotic anaphase, Protein catabolic process	ADAM9, ANAPC1, ANAPC10, ANAPC11, ANAPC16, ANAPC4, ANAPC5, ANAPC7, C11orf51, CCNB1, CDC16, CDC23, CDC26, CDC27, FZR1, HERC5, HSF2, MAD2L1, MAD2L2, PTTG1, PTTG1IP, REV3L, SNX9, UBE2CBP
