# Supplementary material for: Identification of Unstable Network Modules Reveals Disease Modules Associated with the Progression of Alzheimer’s Disease
Source: PLoS One. 2013 Nov 15;8(11):e76162. doi: 10.1371/journal.pone.0076162 (PMC3858171; doi:10.1371/journal.pone.0076162)
Supplement: Table S3 — The list of AD-disrupted modules. The “Enriched GO annotation” column indicates significant cellular functions by an enrichment analysis. The “Gene symbol” column indicates genes expressed once in stages. In cases of early- and late-disrupted type modules, “Gene symbol” shows genes in AD modules that corresponded to modules in normal aging. (PDF) [file pone.0076162.s012.pdf]

	Brain region	Inherited	Appearing	Disappearing	Inherited/Appearing	Inherited/Disappearing
Normal aging	EC	18	11	10	1.64	1.80
	HIP	18	11	11	1.64	1.64
	SFG	19	10	10	1.90	1.90
AD	EC	18	10	10	1.80	1.80
	HIP	22	11	10	2.00	2.20
	SFG	15	10	10	1.50	1.50
